# Sycp1 Is Not Required for Subtelomeric DNA Double-Strand Breaks but Is Required for Homologous Alignment in Zebrafish Spermatocytes

**DOI:** 10.3389/fcell.2021.664377

**Published:** 2021-03-26

**Authors:** Yukiko Imai, Kenji Saito, Kazumasa Takemoto, Fabien Velilla, Toshihiro Kawasaki, Kei-ichiro Ishiguro, Noriyoshi Sakai

**Affiliations:** ^1^Department of Gene Function and Phenomics, National Institute of Genetics, Mishima, Japan; ^2^Department of Genetics, School of Life Sciences, SOKENDAI (The Graduate University for Advanced Studies), Mishima, Japan; ^3^Department of Chromosome Biology, Institute of Molecular Embryology and Genetics, Kumamoto University, Kumamoto, Japan

**Keywords:** Sycp1, meiosis, recombination, zebrafish, synapsis, synaptonemal complex

## Abstract

In meiotic prophase I, homologous chromosomes are bound together by the synaptonemal complex, in which two axial elements are connected by transverse filaments and central element proteins. In human and zebrafish spermatocytes, homologous recombination and assembly of the synaptonemal complex initiate predominantly near telomeres. In mice, synapsis is not required for meiotic double-strand breaks (DSBs) and homolog alignment but is required for DSB repair; however, the interplay of these meiotic events in the context of peritelomeric bias remains unclear. In this study, we identified a premature stop mutation in the zebrafish gene encoding the transverse filament protein Sycp1. In *sycp1* mutant zebrafish spermatocytes, axial elements were formed and paired at chromosome ends between homologs during early to mid-zygonema. However, they did not synapse, and their associations were mostly lost in late zygotene- or pachytene-like stages. In *sycp1* mutant spermatocytes, γH2AX signals were observed, and Dmc1/Rad51 and RPA signals appeared predominantly near telomeres, resembling wild-type phenotypes. We observed persistent localization of Hormad1 along the axis in *sycp1* mutant spermatocytes, while the majority of Iho1 signals appeared and disappeared with kinetics similar to those in wild-type spermatocytes. Notably, persistent Iho1 foci were observed in *spo11* mutant spermatocytes, suggesting that Iho1 dissociation from axes occurs in a DSB-dependent manner. Our results demonstrated that Sycp1 is not required for peritelomeric DSB formation but is necessary for complete pairing of homologs in zebrafish meiosis.

## Introduction

During meiosis, homologous chromosomes (homologs) undergo recombination that generates reciprocal DNA exchanges called crossovers. Since crossovers provide physical connections between homologs, meiotic recombination is essential for faithful segregation of chromosomes in most organisms (Hunter, 2015). This recombination is initiated in meiotic prophase I by the programmed formation of DNA double-strand breaks (DSBs) that are repaired to ensure at least one crossover per homolog pair (Bolcun-Filas and Schimenti, 2012).

Meiotic recombination occurs in the context of a meiosis-specific loop-axis chromatin structure, where arrays of chromatin loops are tethered to a proteinaceous axis (Zickler and Kleckner, 2015). After DSB formation, homologous chromosomes are synapsed along the axis structure (axial element, AE) through the formation of the synaptonemal complex (SC). In the SC, two AEs (now called lateral elements, LEs) spanning the ∼100 nm distance are connected at the central region (CR), which comprises transverse filaments attached within the central element (CE) (Zickler and Kleckner, 2015). The tripartite structure of the SC is largely conserved among eukaryotes, while the amino acid sequences of individual components vary among organisms (Westergaard and von Wettstein, 1972; Grishaeva and Bogdanov, 2014). In mammals, eight SC components have been identified: AE proteins SYCP2 and SYCP3, the transverse filament (TF) protein SYCP1 (Meuwissen et al., 1992), and CE proteins SYCE1, SYCE2, SYCE3, SIX6OS1, and TEX12 (Costa et al., 2005; Hamer et al., 2006; Schramm et al., 2011; Gómez-H et al., 2016). SYCP1 is the main structural element of TFs and self-assembles into a supramolecular zipper-like lattice that mediates synapsis (Schramm et al., 2011; Dunce et al., 2018). The SC is thought to serve as a scaffold for recombination because most of the proteins required for DSB formation and repair localize to it (Baudat et al., 2013).

Meiotic DSB formation is catalyzed by the meiosis-specific endonuclease SPO11, which is widely conserved across eukaryotic lineages (Bergerat et al., 1997; Keeney et al., 1997; Malik et al., 2007), with the help of SPO11 accessory proteins reviewed in de Massy (2013). In mice, REC114, MEI4, IHO1, MEI1, and TOPO6BL, as well as SPO11, are essential for meiotic DSB formation (Keeney et al., 1997; Kumar et al., 2010, 2018, 2; Robert et al., 2016; Stanzione et al., 2016). IHO1 interacts with the axis-associated protein HORMAD1 and recruits REC114 and MEI4 onto axes (Stanzione et al., 2016; Kumar et al., 2018). These proteins are known to be excluded from synapsed axes, and HORMAD1 depletion is dependent on synapsis and the AAA-ATPase TRIP13 (Wojtasz et al., 2009; Kumar et al., 2010, 2018; Stanzione et al., 2016). Thus, synapses could be potentially involved in the regulation of DSB formation by removing DSB-promoting proteins, although the role of SCs remains largely unknown.

In mice, SYCP1 and all CE proteins are crucial for meiotic recombination as well as SC assembly, since their individual knockout leads to meiotic arrest with DSB repair defects (de Vries et al., 2005; Bolcun-Filas et al., 2007, 2009; Hamer et al., 2008; Schramm et al., 2011; Gómez-H et al., 2016). For example, in *Sycp1* knockout mice, AEs assemble normally and align homologously but do not synapse (de Vries et al., 2005). In *Sycp1^–/–^* mouse spermatocytes, γH2AX (the phosphorylated form of histone H2AX that appears in response to DSBs) appears normal in leptonema but remains in a number of discrete domains in pachynema; foci of RAD51, DMC1, and RPA (single-stranded DNA-binding proteins), as well as MSH4 (a MutS homolog protein stabilizing recombination intermediates), appear similarly as in wild-type spermatocytes but do not all disappear, and MLH1 and MLH3 (factors promoting crossover repair) foci are absent (de Vries et al., 2005). These observations indicate that SYCP1 is not required for the global formation of DSBs and for early recombinase recruitment but is necessary for the efficient repair of DSBs as crossovers in mice.

While meiosis has been studied extensively in mice as a mammalian model, differences in key meiotic features have been observed between humans and mice. In mice, meiotic DSBs visualized by single-stranded DNA-binding proteins (such as DMC1, RAD51, or RPA) are observed as a few hundred foci per nucleus that localize along the entire chromosome at both the cytological and sequence levels (Barlow et al., 1997; Mahadevaiah et al., 2001; Smagulova et al., 2011; Brick et al., 2018; Hinch et al., 2020). This chromosome-scale pattern of DSB distribution is similarly observed in both male and female mice (Brick et al., 2018). In humans, chromosome-scale distributions of DSBs in males are specific and strongly skewed toward telomeres (Pratto et al., 2014). The DSB frequency in human males correlates well with the crossover rate, which is also high in subtelomeric regions (Barlow and Hultén, 1998; Kong et al., 2002; Coop et al., 2008; Khil and Camerini-Otero, 2010; Lu et al., 2012; Wang et al., 2012; Kirkness et al., 2013; Pratto et al., 2014). In addition, SC formation also initiates at subtelomeric regions in human males, while in both male and female mice, it often initiates at multiple sites located along the entire chromosome, excluding centromeres (Bisig et al., 2012; Gruhn et al., 2016). Since reverse genetics approaches are difficult in humans, many features of meiotic recombination in the context of subtelomeric bias remain to be elucidated.

Zebrafish (*Danio rerio*) is an emerging model for studying meiotic recombination and has several characteristics similar to those of humans (Sansam and Pezza, 2015; Blokhina et al., 2019; Takemoto et al., 2020). In zebrafish spermatocytes, meiotic DSBs, crossovers, and initiation of homologous pairing and synapsis are predominantly observed near telomeres (Brown et al., 2005; Pratto et al., 2014; Saito et al., 2014; Blokhina et al., 2019; Takemoto et al., 2020). Thus, zebrafish have great potential for use to uncover the mechanisms underlying the subtelomeric bias of meiotic events, which are less obvious in mice. Both forward and reverse genetic approaches are accessible in zebrafish, and several meiotic mutant lines have been isolated (Feitsma et al., 2007; Rodríguez-Marí et al., 2010; Shive et al., 2010; Saito et al., 2011; Blokhina et al., 2019; Takemoto et al., 2020).

We previously isolated three mutant zebrafish lines generated by N-ethyl-N-nitrosourea (ENU) mutagenesis that present male sterile phenotypes (Saito et al., 2011). In this study, we identified the causal mutation of one of these mutant lines, named *iesada* (*isa*), in the *sycp1* gene. In zebrafish, Sycp1 has been reported to be expressed in early primary oocytes and primary spermatocytes and spermatids at the transcriptional level (Gautier et al., 2013) and in primary oocytes and primary spermatocytes at the protein level (Moens, 2006; Kochakpour and Moens, 2008). While Sycp1 staining patterns on zebrafish chromosome spreads suggest its function in meiotic SC formation (Moens, 2006; Kochakpour and Moens, 2008; Saito et al., 2014; Blokhina et al., 2019; Takemoto et al., 2020), its exact roles have not been verified in zebrafish by loss-of-function approaches. Therefore, we further analyzed the meiotic phenotypes of this *sycp1* mutant zebrafish to understand Sycp1 functions and the interplay of meiotic events in the context of subtelomeric bias. Our data demonstrated that homologous pairing occurs only transiently at chromosome ends and cannot be formed over entire chromosomes in *sycp1* mutant zebrafish. Furthermore, meiotic DSBs occur predominantly near telomeres even in the absence of Sycp1, and Hormad1 but not Iho1 remains on axes.

## Results

### A Premature Stop Mutation in *Sycp1* Is Responsible for *isa* Phenotypes

**FIGURE 1:**
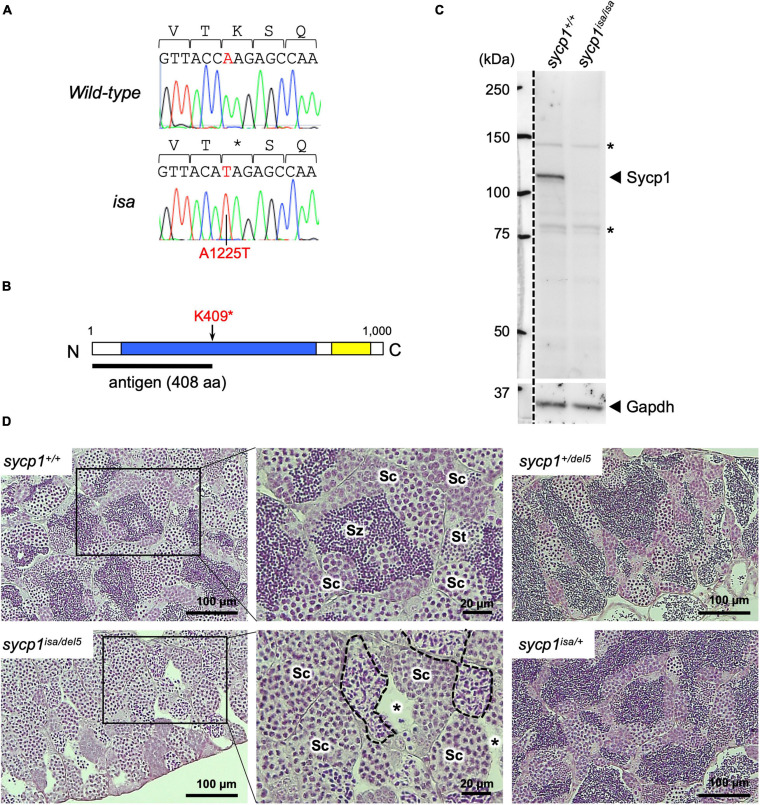
A premature stop mutation of the *sycp1* gene in the *isa* mutant line. **(A)** Genomic sequences of a part of exon 15 in the *sycp1* gene. DNA sequences and peaks obtained from a wild-type and an *isa* mutant fish are shown with corresponding amino acids (above in 1-letter abbreviations). Positions of the *isa* mutation (base 1225 of the *sycp1* coding sequence) are shown in red letters. **(B)** A schematic presentation of the Sycp1 protein sequence. The *isa* mutation site is indicated with an arrow. The antigen region (amino acids 1 to 408) of the Sycp1 antibody used in this study is shown below as a black bar. N: N-terminus, C: C-terminus. Numbers indicate corresponding amino acid positions. **(C)** Western blotting of the Sycp1 protein. Testis protein extracts of wild-type (*sycp1^+/+^)* and *isa* homozygote mutant (*sycp1*^*isa/isa*^) adult fish were blotted with anti-Sycp1 antibody and anti-Gapdh antibody as an internal control. The left side of the broken line is a part labeled with a molecular ladder on the same membrane. The predicted molecular sizes of full-length Sycp1 and Gapdh proteins are 116 kDa and 35.8 kDa, respectively. Non-specific bands were marked with asterisks (*). **(D)** HE-stained sections of *sycp1^+/+^, sycp1^*isa/del5*^, sycp1^*isa/+*^* and *sycp1*^*del5/+*^ testes. All samples were prepared from siblings at 4 mpf (months post fertilization). Representative results of 3 individual fish are shown for each genotype. Magnified images are shown for *sycp1*^+/+^ and *sycp1*^*isa/del5*^ sections. Sc: spermatocytes, St: spermatids, Sz: spermatozoa. In the *sycp1*^*isa/del5*^ section, lumens with no spermatozoa (asterisks) and spermatocytes with irregular nuclei (inside broken lines) were observed.

Previously, we reported ENU-induced mutant zebrafish lines associated with defective spermatogenesis (Saito et al., 2011). One of these mutant lines, called *iesada* (*isa*), was characterized by the accumulation of Sycp3-positive cells and a lack of haploid cells, suggesting defects in meiosis I. To identify the mutation responsible for the *isa* phenotypes, we performed screening with SSLP markers and identified a 544-kbp region containing the *sycp1* gene (see section “Materials and Methods,” Supplementary Figure 1). Since *Sycp1* is known to be essential for the progression of meiotic prophase I in other species (Sym et al., 1993; Zickler and Kleckner, 1999; MacQueen et al., 2002; de Vries et al., 2005; Higgins et al., 2005; Osman et al., 2006; Fraune et al., 2012), we further examined its genomic sequence in *isa* and identified a premature stop mutation at the 1225th base of the coding sequence (A1225T, Figure 1A). This mutation, named *sycp1*^*isa*^, potentially alters the lysine at position 409 to a stop codon and leads to aberrant Sycp1 protein expression (Figure 1B). Therefore, we analyzed the expression of the Sycp1 protein by western blotting, and we detected no Sycp1 protein in *sycp1*^*isa/isa*^ testis extract (Figure 1C). Next, we carried out complementary tests using a *sycp1* knockout allele generated by CRISPR-Cas9 mutagenesis (*sycp1^*del*5^*; see section “Materials and Methods”). The *sycp1^*del*5^* allele harbors a −5 bp frameshift mutation in exon 3 and is predicted to generate a protein of 87 amino-acids, in case of translation. After crossing *sycp1*^*isa/+*^ and *sycp1^*del*5/+^* zebrafish, testis histology of *sycp1*^+/+^, *sycp1*^*isa/+*^, *sycp1^*del*5/+^*, and *sycp1^*isa/del*5^* siblings was compared to examine whether the *sycp1^*del*5^* mutation can complement the spermatogenic defects caused by the *isa* mutation (Figure 1D). Strikingly, an accumulation of spermatocytes with neither spermatids nor spermatozoa was observed in *sycp1^*isa/del*5^* testes (Figure 1D; *sycp1^*isa/del*5^*). We also observed spermatocysts with irregularly condensed nuclei (Figure 1D; broken lines), as observed in *sycp1*^*isa/isa*^ and *sycp1^*del*5/del5^* testes (Supplementary Figure 2) (Saito et al., 2011). These phenotypes were in clear contrast with those of *sycp1*^+/+^, *sycp1*^*isa/+*^, and *sycp1^*del*5/+^* testes, where spermatids and accumulation of spermatozoa in the lumen of lobules were observed (Figure 1D; St and Sz). Therefore, the *sycp1^*del*5^* allele did not complement the *isa* phenotypes. Taken together, we confirmed that the absence of the Sycp1 protein caused by the A1225T non-sense mutation in the *sycp1* gene is responsible for *isa* phenotypes.

### Sycp1 Is Required for the Ovarian Development of Zebrafish

**FIGURE 2:**
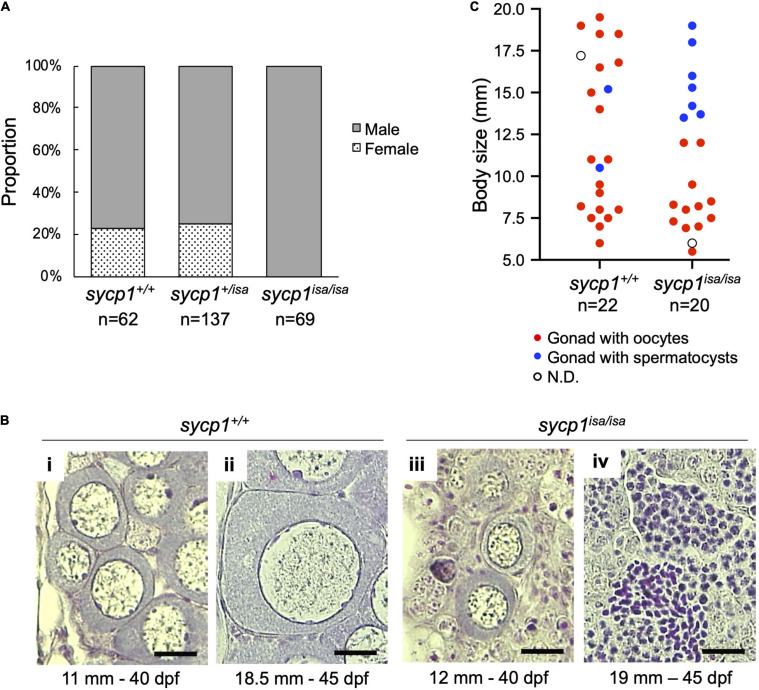
Sexual phenotypes of *sycp1* mutant zebrafish. **(A)** Sexual phenotypes of offspring from *sycp1*^ +/*isa*^ incrosses. Proportions of offspring with testes (males) and ovaries (females) are shown for each genotype. The results of two incross matings were pooled and shown. **(B)** HE-stained sections of juvenile *sycp1*^+/+^ and *sycp1*^*isa/isa*^ gonads. Representative images of *sycp1*^+/+^ (i and ii) and *sycp1*^*isa/isa*^ (iii and iv) gonads with primary oocytes (i to iii) or spermatocysts (iv) are shown. Their body size (mm) and age (dpf: days post fertilization) are indicated below. Scale bars: 20 μm. **(C)** Phenotypes of juvenile *sycp1*^+/+^ and *sycp1*^*isa/isa*^ gonads. Phenotypes of gonads from 24 to 45 dpf of *sycp1*^+/+^ and *sycp1*^*isa/isa*^ fish were examined by HE staining and categorized into gonads with oocytes (red dots), gonads with spermatocysts (blue dots) and N.D. (not determined: open circle) and were plotted by their body size. Pooled results of siblings from two crosses are shown. Representative images are shown in Figure 2B. Histological images of all analyzed samples are shown in Supplementary Figure 3.

We previously observed male-specific infertility among siblings obtained from intercrossing of *isa* heterozygote male and female zebrafish (Saito et al., 2011). This suggests that *sycp1* mutant females are fertile or that all mutants become male, similar to the case for other reported zebrafish mutants with germline defects (Slanchev et al., 2005; Draper et al., 2007; Houwing et al., 2007; Siegfried and Nüsslein-Volhard, 2008; Kamminga et al., 2010; Rodríguez-Marí et al., 2010; Shive et al., 2010; Takemoto et al., 2020). To examine whether females appear among *sycp1*^*isa/isa*^ zebrafish, we analyzed the *sycp1* genotypes and sexual phenotypes of male and female offspring obtained from the intercrossing of *sycp1*^ +/*isa*^ fish (Figure 2A). Sexual phenotypes were determined by the morphology of adult gonads (≥4 months post fertilization) after dissection. While we detected male fish in all *sycp1*^+/+^, *sycp1*^ +/*isa*^, and *sycp1*^*isa/isa*^ fish (77%, 75% and 100% of each genotype, respectively), female fish were found only among *sycp1*^+/+^ and *sycp1*^ +/*isa*^ fish (23% and 25% of each genotype, respectively). Since zebrafish sex is genetically determined with limited and secondary influences from the environment (Liew et al., 2012), the sex ratios could vary from 1:1 even in wild-type contexts. This result indicates that all *sycp1*^*isa/isa*^ zebrafish developed as males. In zebrafish gametogenesis, all juvenile gonads first generate immature oocytes regardless of their definitive sex, and the individuals in which these immature oocytes degenerate develop as males (Uchida et al., 2002; Maack and Segner, 2003; Rodríguez-Marí et al., 2005). Thus, the absence of females in *sycp1*^*isa/isa*^ fish (Figure 2A) implies that oocytes were eliminated during gametogenesis, most likely at the juvenile stage. To determine the stages of oocyte degeneration, we compared the histology of *sycp1*^+/+^ and *sycp1*^*isa/isa*^ gonads from 24 to 45 days post fertilization (dpf) (Figures 2B,C, Supplementary Figure 3). Since differences in environmental conditions affect the development of juvenile fish and lead to variations in zebrafish growth and gonad development (Elkouby and Mullins, 2017), we also measured the body sizes of the sampled fish. Gonads with previtellogenic dictyate (stage IB) (Selman et al., 1993) oocytes were found in both *sycp1*^+/+^ and *sycp1*^*isa/isa*^ fish (Figure 2B; images of all analyzed gonads are in Supplementary Figure 3). While gonads with oocytes up to ∼40 μm in diameter were found in both *sycp1*^*isa/isa*^ and *sycp1*^+/+^ fish (Figure 2Bi, iii), gonads with oocytes larger than 40 μm were observed only among *sycp1*^+/+^ fish (Figure 2Bii). In relatively large juvenile *sycp1*^*isa/isa*^ fish (body size more than ∼13.5 mm), only gonads with spermatocysts were observed (Figures 2Biv, C, Supplementary Figure 3). These observations support the hypothesis that oocytes appear in juvenile *sycp1*^*isa/isa*^ gonads but are lost during stage IB, and all *sycp1*^*isa/isa*^ fish develop to males (Figure 2A).

### Sycp1 Is Not Required for Axis Formation in Zebrafish Spermatocytes

**FIGURE 3:**
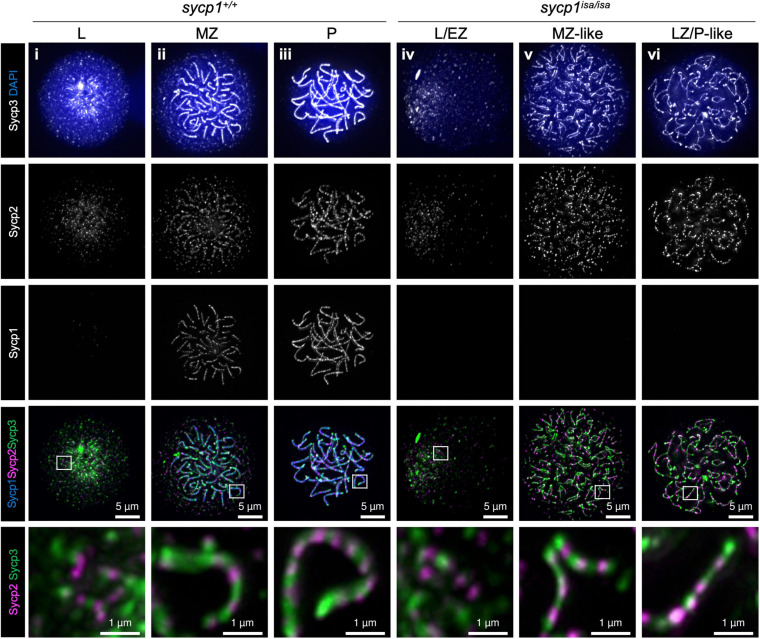
Formation of chromosomal axes in *sycp1* mutant spermatocytes. Immunostaining of Sycp3, Sycp2 and Sycp1 components in wild-type (*sycp1*^+/+^, i to iii) and *sycp1*^*isa/isa*^ (iv to vii) spermatocyte chromosome spreads. Wild-type nuclei at leptotene (L, i), mid-zygotene (MZ, ii) and pachytene (P, iii) were staged according to the staining patterns of Sycp3, Sycp2 and Sycp1 (Blokhina et al., 2019). *sycp1*^*isa/isa*^ nuclei at leptotene (L, iv), mid-zygotene-like (MZ-like, v) and late zygotene/pachytene-like (LZ/P-like, vi) stages were staged by axis staining patterns. Regions indicated in white rectangles are shown at the bottom at a higher magnification.

The primary function of mammalian SYCP1 is to connect homologous chromosomal axes as a transverse filament component of the synaptonemal complex (de Vries et al., 2005). To examine whether Sycp1 has a conserved function in zebrafish, we analyzed the meiotic phenotypes of the *sycp1* mutant by immunostaining of spermatocyte spreads. First, we examined axis formation by costaining of axial elements Sycp2 and Sycp3, together with Sycp1, on wild-type (*sycp1*^+/+^) and *sycp1* mutant (*sycp1*^*isa/isa*^) spermatocytes (Figure 3). In *sycp1*^+/+^ spermatocytes, Sycp2 and Sycp3 start to form short axis fragments together at leptonema (Figure 3i). This axis formation initiates near chromosome ends clustered as a telomere bouquet and progresses inwards (Saito et al., 2014; Blokhina et al., 2019; Takemoto et al., 2020, see below for telomere staining). At zygonema, Sycp1 appears as short fragments, colocalizing with Sycp2 and Sycp3 signals (Figure 3ii), and the entire length of homologous chromosomes was stained for these three proteins in pachynema (Figure 3iii), as we reported previously (Takemoto et al., 2020). Along the progression of zygonema, telomeres disperse from the bouquet (Figure 3ii, see below for telomere staining), and the bouquet structure is no longer evident in pachynema (Figure 3iii; Saito et al., 2014; Blokhina et al., 2019). We also marked that Sycp2 was not evenly stained along axes but rather appeared as discontinuous lines (Figure 3i–iii, bottom images). In *sycp1*^*isa/isa*^ spermatocytes, no Sycp1 signals were observed (Figure 3iv–vi), as expected given the absence of Sycp1 protein by western blotting (Figure 1C). We observed Sycp2/Sycp3-stained axes in leptotene *sycp1*^*isa/isa*^ spermatocytes that appeared similar to those of wild-type spermatocytes (Figure 3iv), as well as their extension in zygotene-like stages (Figure 3v,vi). Therefore, we staged *sycp1*^*isa/isa*^ spermatocytes to preleptotene (PL, no axis), leptotene or early zygotene-like (L/EZ-like, punctate or short axes in a cluster), mid-zygotene-like (MZ-like, longer axes than L/EZ-like nuclei dissociating from a bouquet), or late zygotene/pachytene-like (LZ/P-like, axes similar to wild-type pachytene nuclei and axes ends are dispersed over nuclei) stages according to their axis staining patterns. Notably, we observed associations of two Sycp2-/Sycp3-stained axes in MZ-like *sycp1*^*isa/isa*^ spermatocytes (Figure 3v, the bottom image). These associations were largely lost in LZ/P-like stages, and only a few paired ends were observed (Figure 3vi). Sycp2 was observed as discontinuous lines in *sycp1*^*isa/isa*^ spermatocytes (Figure 3iv–vi, bottom images), similar to wild-type spermatocytes (Figure 3i–iii, bottom images). Thus, we concluded that axis formation occurs in the absence of Sycp1 in zebrafish spermatocytes.

### Sycp1 Is Required for Complete Homolog Alignment in Zebrafish Spermatocytes

**FIGURE 4:**
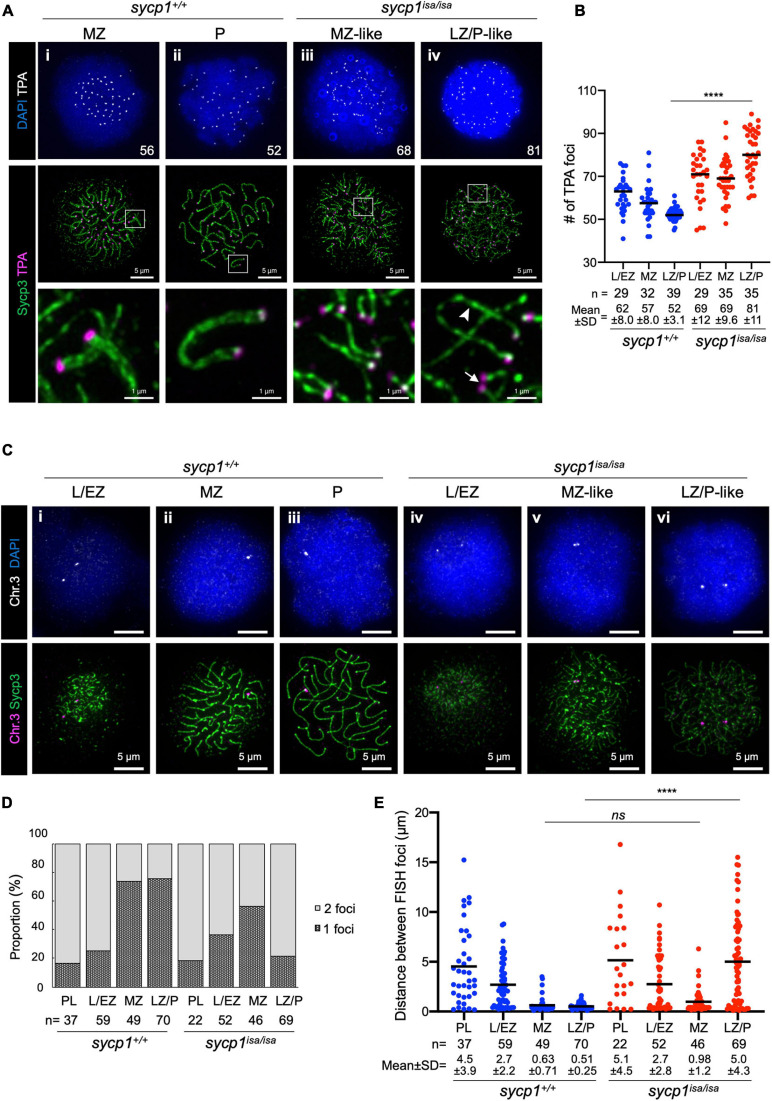
Transient pairing of homologs at chromosome ends in *sycp1* mutant spermatocytes. **(A)** costaining of telomeres (TPA) and Sycp3 in *sycp1*^+/+^ (i and ii) and *sycp1*^*isa/isa*^ (iii and iv) spermatocytes. The arrowhead indicates complete axis formation between two telomeres, and the arrow indicates paired telomere foci in LZ/P-like *sycp1*^*isa/isa*^ spermatocytes. Numbers in the top panels indicate counts of TPA foci. Regions indicated in white rectangles are shown at a higher magnification at the bottom. **(B)** quantification of telomere polyamide (TPA) foci in *sycp1*^+/+^ and *sycp1*^*isa/isa*^ spermatocytes. Black bars indicate means. Statistical significance was examined by a two-tailed Mann-Whitney test (****P < 0.0001; exact *P* value). **(C)** fish staining of a peritelomeric locus on chromosome 3 in *sycp1*^+/+^ (i to iii) and *sycp1*^*isa/isa*^ (iv to vi) spermatocytes. **(D)** Proportion of nuclei with one or two FISH foci in *sycp1*^+/+^ and *sycp1*^*isa/isa*^ spermatocytes. **(E)** quantification of distances between FISH foci of a peritelomeric locus on chromosome 3 in *sycp1*^+/+^ and *sycp1*^*isa/isa*^ spermatocytes. Distances between FISH foci observed in Figure 4D. For nuclei with only one FISH focus, the radius of the FISH-stained area was considered the distance between two FISH foci. Black bars indicate means. Statistical significance was examined by a two-tailed Mann-Whitney test (****P < 0.0001, ns, not significant; exact *P* value).

In *sycp1*^*isa/isa*^ spermatocytes, we observed associations of two chromosomal axes in MZ-like stages (Figure 3v). Since axis formation preferentially initiates near telomeres in zebrafish (Saito et al., 2014; Blokhina et al., 2019; Takemoto et al., 2020), we asked whether this pairing occurs near telomeres. To examine this, we visualized telomeres by telomere polyamide (TPA), a pyrrole–imidazole polyamide probe that binds to telomere sequences (Sasaki et al., 2016). After costaining of Sycp3 and telomeres by TPA, we found that axes emanated from telomeres, as observed in wild-type spermatocytes (Figure 4Ai), even in the absence of Sycp1 (Figure 4Aiii). In LZ/P-like *sycp1*^*isa/isa*^ spermatocytes, we observed complete axis formation from telomeres to telomeres with some chromosomes (Figure 4Aiv, arrowhead). Furthermore, the associations of two Sycp3-stained axes occurred in proximity to TPA-stained foci in MZ-like *sycp1*^*isa/isa*^ spermatocytes (Figure 4Aiii, the bottom image), indicating that the axes paired near telomeres even in the absence of Sycp1. Such pairing was observed with a few axes in later LZ/P-like stages (Figure 4Aiv, arrow). To evaluate the pairing of chromosome ends, we compared the numbers of TPA foci in *sycp1*^+/+^ and *sycp1*^*isa/isa*^ spermatocytes (Figure 4B). Because zebrafish have 25 pairs of homologous chromosomes, 50 telomere foci are expected after tight axis associations by complete pairing. Consistent with this, 52 ± 3.1 (mean ± SD) foci were observed in LZ/P *sycp1*^+/+^ spermatocytes, whereas far more than 50 foci were observed in L/EZ (62 ± 8.0) and MZ (57 ± 8.0) spermatocytes, because some homologs have yet to be paired at these stages (Figure 4B). In *sycp1*^*isa/isa*^ spermatocytes, we observed more than 50 TPA foci in most spermatocytes at L/EZ-like (69 ± 12), MZ-like (69 ± 9.6) and LZ/P-like (81 ± 11) spermatocytes (Figure 4B), though at MZ-like stages, most TPA foci were observed in pairs (Figure 4Aiii). The number of TPA foci in LZ/P-like *sycp1*^*isa/isa*^ spermatocytes was significantly higher than that in wild-type LZ/P spermatocytes (Figure 4B). These data indicate that tight associations between homologous axes at chromosome ends were lost in the absence of Sycp1. However, it should be noted that non-homologous associations of telomeres occur independently of DSB in zebrafish (Blokhina et al., 2019). In addition, paired but not tightly associated telomeres, as observed in MZ-like *sycp1*^*isa/isa*^ spermatocytes, cannot be evaluated by TPA quantification.

To further examine whether pairing of axis observed in MZ-like *sycp1*^*isa/isa*^ spermatocytes (Figure 3Av, 4Aiii) occurred between homologs, we performed DNA fluorescence *in situ* hybridization (DNA FISH, Figures 4C–E). To assess homologous pairing, we used a DNA probe specific to a 35.9-kbp region close to a telomere on chromosome 3 (see section “Materials and Methods”). In wild-type spermatocytes, FISH foci were observed as two foci in the majority of early prophase I cells (Figure 4Ci; 84% and 75% of PL and L/EZ cells, respectively; Figure 4D) and as one bright focus at the end of the Sycp3-stained axis in the majority of MZ (Figure 4Cii) and LZ/P (Figure 4Ciii) cells (73% and 76%, respectively; Figure 4D). This indicates that the homologous sites at the end of chromosome 3 became closer during the progression of prophase I and remained paired throughout zygonema and pachynema in wild-type spermatocytes (Figure 4E). In *sycp1*^*isa/isa*^ spermatocytes, we observed two foci in 82% and 63% of PL and L/EZ cells, respectively (Figure 4Civ, D), similar to the case in wild-type spermatocytes. Strikingly, we observed one or two closely paired foci in most MZ-like *sycp1*^*isa/isa*^ spermatocytes (Figure 4Cv, D), and the distances between the two FISH foci in those cells were not significantly different from those in wild-type MZ spermatocytes (0.98 ± 1.2 and 0.63 ± 0.71 in *sycp1*^*isa/isa*^ and wild-type MZ spermatocytes, respectively; Figure 4E). In contrast, in the LZ/P-like stages, 78% of *sycp1*^*isa/isa*^ spermatocytes showed two FISH foci (Figures 4Cvi, D), and the distances between FISH foci were significantly higher than those in wild-type LZ/P spermatocytes (5.0 ± 4.3 and 0.51 ± 0.25, respectively; Figure 4E). These observations indicate that chromosome 3 paired at the probed site even in the absence of Sycp1, but this homologous association was mostly lost at LZ/P-like stages. Taken together, these data support the idea that homologous pairing of chromosome ends occurs but is not maintained in the absence of Sycp1.

### Sycp1 Is Not Required for DSB Formation in Zebrafish Spermatocytes

**FIGURE 5:**
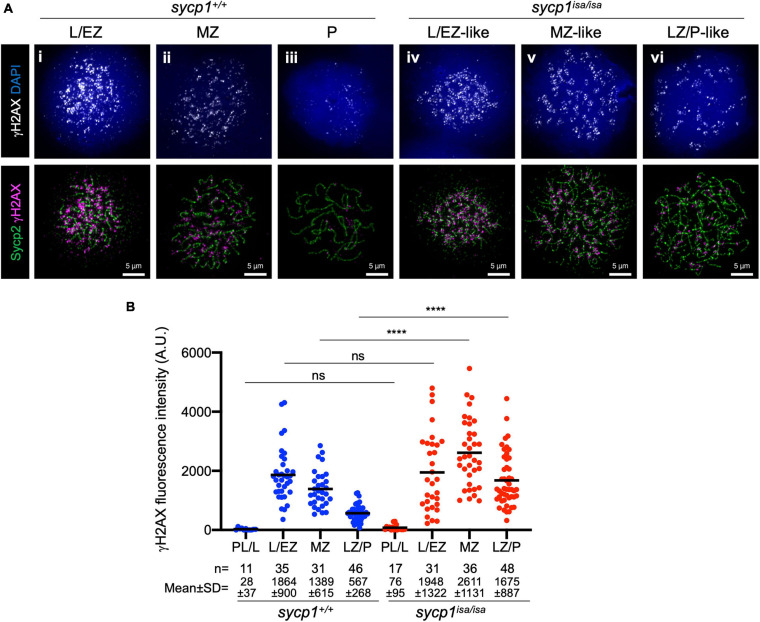
Meiotic DSB formation in *sycp1* mutant spermatocytes. **(A)** costaining of γH2AX and Sycp2 in *sycp1*^+/+^ (i to iii) and *sycp1*^*isa/isa*^ (iv to vi) spermatocytes. **(B)** quantification of γH2AX signal intensity in *sycp1*^+/+^ and *sycp1*^*isa/isa*^ spermatocytes. Black bars indicate means. Statistical significance was examined by a two-tailed Mann-Whitney test (****P < 0.0001, ns, not significant; exact *P* value).

Meiotic recombination is initiated with programmed DSBs (Baudat et al., 2013). To determine whether DSBs occur in *sycp1* mutant zebrafish, we examined γH2AX signals in *sycp1*^+/+^ and *sycp1*^*isa/isa*^ spermatocytes (Figure 5). γH2AX is a phosphorylated form of the histone variant H2AX, which appears in response to DSB formation (Scully and Xie, 2013). This histone mark is also observed in zebrafish meiosis, depending on the Spo11 nuclease (Saito et al., 2011; Blokhina et al., 2019; Takemoto et al., 2020). Consistent with previous reports, γH2AX signals appeared at the leptotene to EZ stages and diminished during zygonema. The majority of γH2AX signals disappeared, and only weak signals remained at pachynema in wild-type spermatocytes (Figures 5Ai–iii, B). In *sycp1*^*isa/isa*^ spermatocytes, γH2AX signals appeared at leptotene to L/EZ-like stages (Figure 5Aiv), similar to the case in wild-type spermatocytes. However, γH2AX signals remained at significantly higher levels in MZ-like and LZ/P-like *sycp1*^*isa/isa*^ spermatocytes (Figure 5Av, vi) than in wild-type MZ and LZ/P cells (Figure 5B). These results suggest that in the absence of Sycp1, meiotic DSBs occur but are not efficiently repaired and/or are initiated continuously in late prophase I.

### DSBs Predominantly Occur at Subtelomeric Regions in *sycp1^*isa/isa*^* Zebrafish Spermatocytes

**FIGURE 6:**
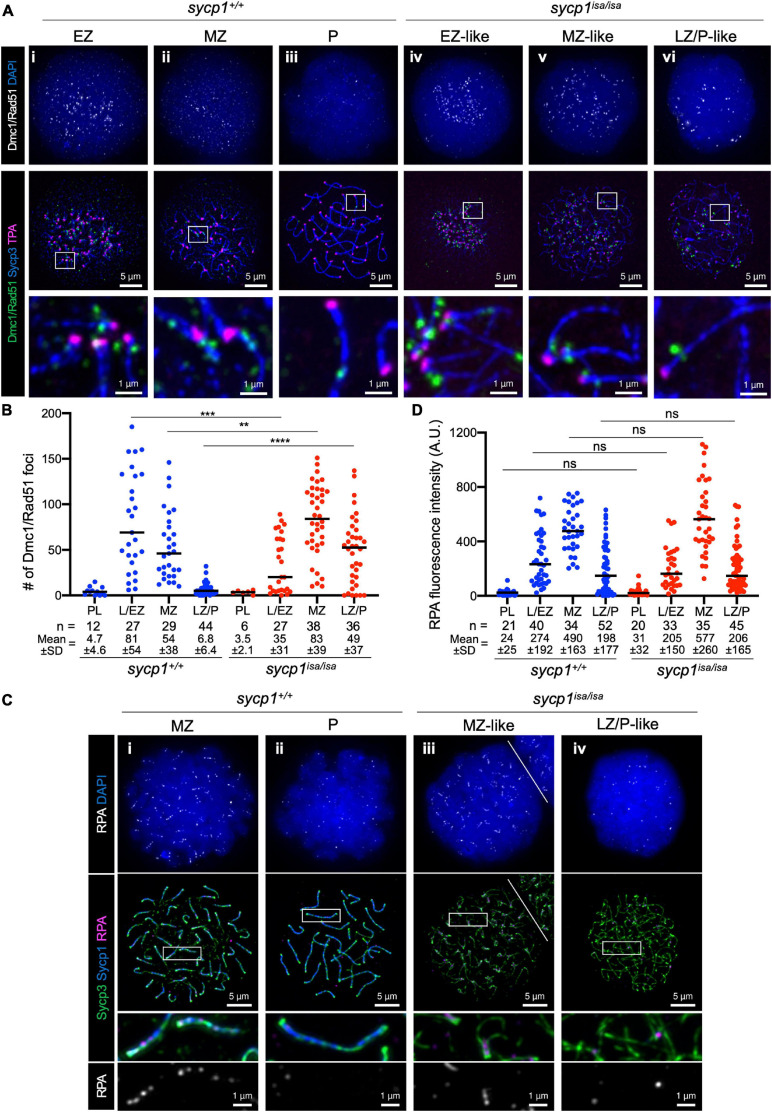
DSB localization in *sycp1* mutant spermatocytes. **(A)** costaining of Dmc1/Rad51, telomeres and Sycp3 in *sycp1*^+/+^ (i to iii) and *sycp1*^*isa/isa*^ (iv to vi) spermatocytes. **(B)** quantification of Dmc1/Rad51 focus numbers in *sycp1*^+/+^ and *sycp1*^*isa/isa*^ spermatocytes. Black bars indicate means. Statistical significance was examined by a two-tailed Mann-Whitney test (**P < 0.01, ***P < 0.001, ****P < 0.0001; exact *P* value). **(C)** costaining of RPA, Sycp1 and Sycp3 in *sycp1*^+/+^ (i and ii) and *sycp1*^*isa/isa*^ (iii and iv) spermatocytes. Regions marked as a white rectangle in the middle panels are shown at a higher magnification at the bottom. The white line on (iii) indicates a border with another nucleus on the top right. **(D)** quantification of RPA signal intensity in *sycp1*^+/+^ and *sycp1*^*isa/isa*^ spermatocytes. Black bars indicate means. Statistical significance was examined by two-tailed Mann-Whitney test (ns, not significant; exact *P* value). Since RPA is observed as short stretches rather than discrete foci, signal intensity rather than the number of foci in a nucleus was quantified here. Quantification of RPA focus numbers is shown in Supplementary Figure 5.

To further analyze early meiotic recombination in *sycp1* mutant fish, we examined the localization of RecA homolog proteins that bind to single-stranded DNAs during DSB repair (Gray and Cohen, 2016). We first performed immunostaining using an anti-Dmc1/Rad51 antibody raised against zebrafish Dmc1 (Figures 6A,B; Takemoto et al., 2020). In *sycp1*^+/+^ spermatocytes, Dmc1/Rad51 foci appeared mostly near telomeres in L/EZ spermatocytes (Figure 6Ai). Their numbers were reduced during zygonema (Figure 6Aii), and only a few or no Dmc1/Rad51 foci were observed at the LZ/P stages in *sycp1*^+/+^ spermatocytes (Figures 6Aiii, B), as observed previously (Sansam and Pezza, 2015; Blokhina et al., 2019; Takemoto et al., 2020). In *sycp1*^*isa/isa*^ spermatocytes, Dmc1 foci appeared at the L/EZ stages (Figure 6Aiv) at lower numbers than in *sycp1*^+/+^ spermatocytes (Figure 6B). The number of Dmc1/Rad51 foci reached the maximum level at MZ-like stages in *sycp1*^*isa/isa*^ spermatocytes (Figures 6Av, B), and the number of Dmc1/Rad51 foci remained significantly higher at LZ/P-like stages (Figure 6Avi) than in wild-type LZ/P spermatocytes (Figure 6B). Notably, a majority of Dmc1/Rad51 foci in *sycp1*^*isa/isa*^ spermatocytes were located near telomeres (Figure 6Aiv–vi). We counted Dmc1/Rad51 foci in telomere-proximal regions (the 1 μm of axis adjacent to the TPA focus, see section “Materials and Methods”), and found that 65 ± 13% of Dmc1/Rad51 foci were in telomere-proximal regions in MZ-like *sycp1*^*isa/isa*^ spermatocytes (Supplementary Figure 4). This result suggests that DSBs are limited to canonical sites even in the absence of Sycp1.

We also examined the localization of RPA (the replication protein A complex), which is required for the recruitment of DMC1/RAD51 and thus for strand invasion as well as efficient crossover formation during meiotic recombination (Figures 6C,D; Sugiyama et al., 1997; Soustelle et al., 2002; Shi et al., 2019; Hinch et al., 2020). In zebrafish spermatocytes, RPA appears as punctate foci at leptonema and forms short stretches mostly at chromosome ends in zygonema (Moens, 2006; Takemoto et al., 2020). We observed such localization of RPA in both wild-type MZ and *sycp1*^*isa/isa*^ MZ-like spermatocytes (Figure 6Ci, iii), and these signals decreased in wild-type LZ/P spermatocytes (Figure 6Bii) and in LZ/P-like *sycp1*^*isa/isa*^ spermatocytes (Figure 6Biv). No significant difference was observed between the RPA signal intensities of wild-type and *sycp1*^*isa/isa*^ spermatocytes at any stage (Figure 6D), although the number of RPA foci in MZ-like *sycp1*^*isa/isa*^ spermatocytes was slightly lower than that in wild-type MZ spermatocytes (Supplementary Figure 5). Taken together, these results demonstrated that DSBs preferentially occur near telomeres in the absence of Sycp1 and that RPA and Dmc1/Rad51 are recruited to those DSB sites.

### Depletion of Sycp1 Differentially Affects Hormad1 and Iho1 Localization on Chromosomal Axes

**FIGURE 7:**
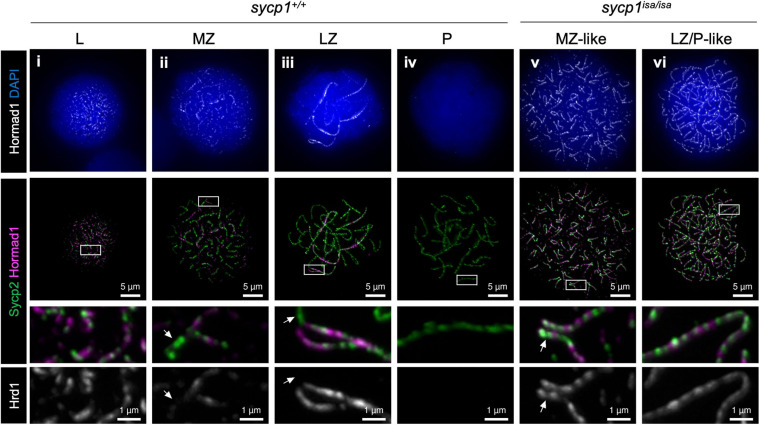
Hormad1 localization in *sycp1* mutant spermatocytes. Costaining of Hormad1 and Sycp2 in *sycp1*^+/+^ **(i to iv)** and *sycp1*^*isa/isa*^ **(v and vi)** spermatocytes. Regions marked as a white rectangle are shown at a higher magnification at the bottom. Arrows indicate synapsed (*sycp1*^+/+^) and paired (*sycp1*^*isa/isa*^) regions of axes.

In mice, proteins promoting DSB formation, such as MEI4, REC114, IHO1, ANKRD31 and HORMAD1, are known to be absent from synapsed axes, implicating that synapsis is involved in DSB regulation (Wojtasz et al., 2009; Fukuda et al., 2010; Kumar et al., 2010; Shin et al., 2010; Stanzione et al., 2016; Boekhout et al., 2019; Papanikos et al., 2019). In *sycp1*^*isa/isa*^ zebrafish, DSBs were still observed at canonical sites near telomeres by Dmc1/Rad51 and RPA staining (Figure 6). To examine the localization of DSB-promoting proteins in the *sycp1* mutant zebrafish, we generated antibodies against zebrafish Hormad1 and Iho1 (see section “Materials and Methods,” Supplementary Figure 6). In mice, HORMAD1 is known as an axis-localizing protein that is required for robust recruitment of IHO1, which in turn recruits MEI4 and REC114 to unsynapsed axes (Stanzione et al., 2016). In wild-type zebrafish spermatocytes, we observed punctate and short filamentous Hormad1 signals partially colocalizing with Sycp2-stained axes at leptonema (Figure 7i). Hormad1 was costained with unsynapsed axes during zygonema (Figure 7ii, iii) and disappeared at pachynema upon complete synapsis of homologs (Figure 7iv). In *sycp1*^*isa/isa*^ spermatocytes, however, we observed persistent localization of Hormad1 on axes at the MZ-like and LZ/P-like stages (Figure 7v, iv). Such Hormad1 localization on unsynapsed axes is similar to what has been observed in mice (Wojtasz et al., 2009; Fukuda et al., 2010; Shin et al., 2010). Notably, Hormad1 was also localized on paired regions of axes that were observed in EZ- to MZ-like *sycp1*^*isa/isa*^ spermatocytes (Figure 7v, arrows). These results indicate that Sycp1 is required for the dissociation of Hormad1 from axes at late prophase I.

**FIGURE 8:**
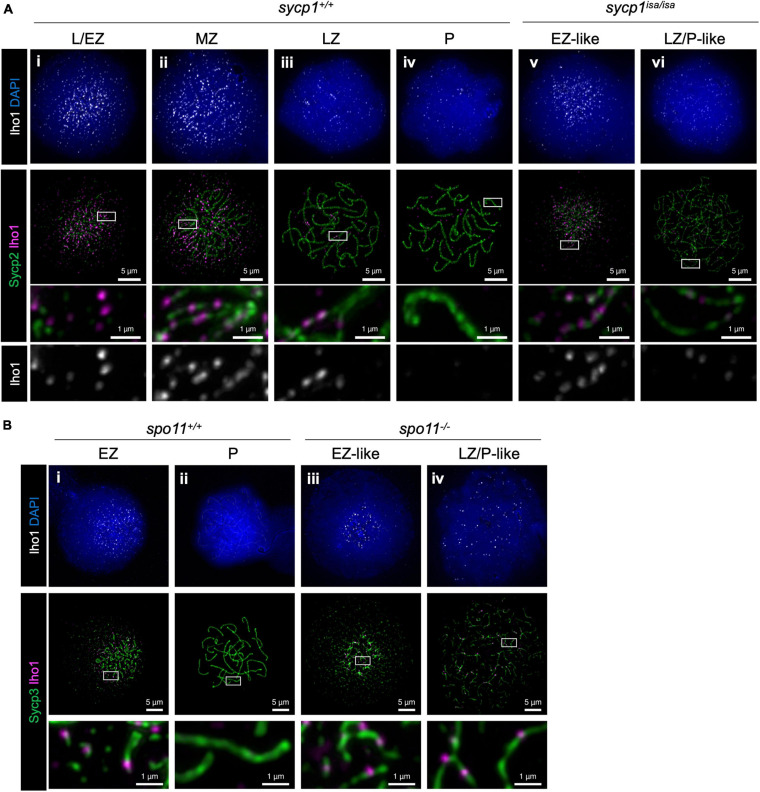
Iho1 localization in *sycp1* and *spo11 m*utant spermatocytes. **(A)** costaining of Iho1 and Sycp2 in *sycp1*^+/+^ (i to iv) and *sycp1*^*isa/isa*^ (v and vi) spermatocytes. Regions marked in white rectangles are shown at a higher magnification at the bottom. **(B)** Costaining of Iho1 and Sycp3 in *spo11*^+/+^ (i and ii) and *spo11*^–/–^ (iii and iv) spermatocytes. Regions marked in white rectangles are shown at a higher magnification at the bottom.

We next analyzed the localization of Iho1 (Figure 8A), the mouse ortholog of which is essential for meiotic DSB formation (Stanzione et al., 2016). In wild-type zebrafish spermatocytes, Iho1 foci were observed on axes at leptonema and zygonema (Figure 8Ai–iii). In the MZ and LZ stages, Iho1 localized on unsynapsed axes as punctuate foci or short stretches (Figure 8Aii, iii) and disappeared from axes at pachynema (Figure 8Aiv). Similarly, we observed bright Iho1 foci in leptotene and EZ-like *sycp1*^*isa/isa*^ spermatocytes (Figure 8Av). These bright signals mostly disappeared in the LZ/P-like stage, and only a few weak signals were observed on some parts of the axes (Figure 8Avi). This was in contrast to Hormad1, in which strong signals persisted on the axes of LZ/P-like *sycp1*^*isa/isa*^ spermatocytes (Figure 7vi). Since bright Iho1 signals disappear even in the absence of Sycp1, we suspected that Iho1 dissociation from axes might be promoted not by synapsis but rather by DSB formation. Thus, we analyzed Iho1 localization in *spo11*^–/–^ spermatocytes that cannot form meiotic DSBs (Blokhina et al., 2019; Takemoto et al., 2020) and observed bright foci of Iho1 in both EZ-like and LZ/P-like *spo11*^–/–^ spermatocytes (Figure 8Biii, iv). This indicates that Iho1 remains associated with distinct sites on axes in the absence of meiotic DSBs. Altogether, our results demonstrated that the spatiotemporal localizations of Iho1 and Hormad1 are differentially affected by depletion of Sycp1.

## Discussion

### Isolation of a *Sycp1* Null Mutant Zebrafish

In this study, we mapped the causal mutation of the *isa* mutant zebrafish line in the *sycp1* gene. Zebrafish *sycp1* is expressed in early primary oocytes and primary spermatocytes and spermatids at the transcriptional level (Gautier et al., 2013) and in primary oocytes and primary spermatocytes at the protein level (Moens, 2006; Kochakpour and Moens, 2008). While zebrafish Sycp1 is known to localize to meiotic homologous chromosomes, as observed in other species (Moens, 2006; Kochakpour and Moens, 2008; Saito et al., 2014; Blokhina et al., 2019; Takemoto et al., 2020), its functions in meiosis have not been elucidated. The *isa* mutation appeared to be a non-sense mutation of the lysine at position 408 in Sycp1, which is 1000 amino acids at full length (Figures 1A,B). Although this mutation is located in the middle of the Sycp1 protein sequence, we did not detect truncated proteins of the expected size (47.7 kDa, Figure 1C). The absence of truncated Sycp1 protein could be explained by non-sense-mediated mRNA decay (NMD), by which mRNAs that contain a premature stop codon located more than 50 nucleotides upstream of the final exon are eliminated (Lewis et al., 2003; Baker and Parker, 2004). In support of this hypothesis, the *isa* mutation is 1632 nucleotides upstream of exon 31, the final exon of *sycp1.* We confirmed by complementation tests that the *sycp1* mutation is responsible for previously reported *isa* phenotypes: accumulation of spermatocytes and absence of spermatids and sperm (Figure 1D; Saito et al., 2011). Altogether, *isa* appeared to be a *sycp1* null mutant zebrafish that is suitable for analyzing the functions and roles of Sycp1 in synapsis in zebrafish meiosis.

### Interdependency of Homologous Pairing, Recombination and Synapsis in Zebrafish Spermatocytes

A recent study in *spo11*-knockout zebrafish indicated that DSBs induced by Spo11 are required for the coalignment of axes and SC formation (Blokhina et al., 2019). Together with previous findings, our data provide evidence to clarify how key meiotic events—homologous pairing, recombination and synapsis—affect each other in zebrafish spermatocytes. In this study, we observed DSB formation in *sycp1* mutant spermatocytes, as indicated by γH2AX, Dmc1/Rad51 and RPA signals (Figures 5, 6). Furthermore, local alignment of homologous axes was observed near telomeres in *sycp1* mutant spermatocytes (Figures 3Avii, 4Bv). These results demonstrated that DSB formation and homologous pairing occur independently of synapsis. However, homologous associations at chromosome ends were largely lost in LZ/P-like stages in the absence of Sycp1 (Figures 3Avi, 4Cvi, D,E). This is in clear contrast to what is observed in *Sycp1* and CE mutant mice, where most homologs remain aligned via parts of the chromosome at pachytene-like stages (Bolcun-Filas et al., 2007, 2009; Hamer et al., 2008; Schramm et al., 2011; de Vries et al., 2014; Gómez-H et al., 2016). This difference in pairing phenotypes might come from the difference between zebrafish and mouse DSB locations. In zebrafish spermatocytes, meiotic DSB formation is heavily biased toward the chromosome ends (Blokhina et al., 2019; Takemoto et al., 2020; Figure 6Ai). In mouse spermatocytes, such peritelomeric bias is not evident (Brick et al., 2018), and the majority of DSBs occur at PRDM9-bound sites across chromosomes (Brick et al., 2012). In *Sycp1* mutant mice, foci of DSB repair proteins (RAD51, RPA and MSH4) are also distributed across the chromosomes (de Vries et al., 2005). Given that DSBs cluster at chromosome ends in zebrafish spermatocytes, homologous interactions at DSB sites (see below) might be unable to provide enough force to support homolog associations and full homolog alignment in the absence of synapsis. Alternatively, most DSBs at aligned axes might be repaired without crossovers, after which homolog associations are resolved at LZ/P-like stages in *sycp1*^*isa/isa*^ zebrafish spermatocytes.

### Meiotic Recombination Defects in *Sycp1* Mutant Zebrafish Spermatocytes

While meiotic DSB formation was observed in *sycp1* mutant zebrafish spermatocytes, these DSBs were unlikely to be repaired properly. In *sycp1* mutant zebrafish spermatocytes, γH2AX signals appeared at L/EZ-like stages, similar to wild-type spermatocytes, but peaked later, at MZ-like stages, and remained strong at LZ/P-like stages (Figure 5B). Accordingly, we observed delayed appearance of Dmc1/Rad51 foci in *sycp1*^*isa/isa*^ spermatocytes compared to wild-type spermatocytes, and many Dmc1/Rad51 foci remained at LZ/P-like stages (Figure 6B). The kinetics of γH2AX and Dmc1/Rad51 signals suggest that early DSB repair is delayed in *sycp1*^*isa/isa*^ spermatocytes (Figures 5B, 6B), similar to what has been observed in *Sycp1* mutant mice (de Vries et al., 2005). In contrast, we observed similar kinetics in RPA intensities between wild-type and *sycp1*^*isa/isa*^ spermatocytes (Figures 6C,D). Recent studies in mice revealed that RPA has dual functions: recruiting DMC1 and RAD51 to exposed ssDNA at DSB sites and binding to the repair template in a D-loop recombination intermediate (Shi et al., 2019; Hinch et al., 2020). In wild-type zebrafish spermatocytes, some RPA foci are observed as short stretches in mid-zygonema (Moens, 2006; Takemoto et al., 2020; Figure 6Ci). These RPA signals may reflect localization on the repair template strand. We speculate that in *sycp1*^*isa/isa*^ spermatocytes, RPA could accumulate at DSB sites because of inefficient loading of Dmc1/Rad51, but for the same reason, its binding to the repair template could be reduced. We might observe the sum of these two different effects which results in no apparent change in RPA signal intensities in *sycp1*^*isa/isa*^ spermatocytes (Figure 6D).

Importantly, we observed short stretches of RPA between aligned axes in MZ-like *sycp1*^*isa/isa*^ spermatocytes (Figure 6Ciii), suggesting that a substantial portion of DSB sites undergo the strand invasion step and form recombination intermediates (Baudat et al., 2013; Brown and Bishop, 2015; Hunter, 2015). This is also supported by our observation that axis alignment at chromosome ends occurs between homologs (Figures 4C–E). The majority of these recombination intermediates are unlikely to form crossovers, since homolog associations at chromosome ends were largely lost in LZ/P-like *sycp1*^*isa/isa*^ spermatocytes (Figures 4D,E). It is possible that those intermediates could be resolved without crossovers (non-crossovers). A minor fraction of intermediates might remain unresolved, since we detected several Dmc1/Rad51 and RPA foci in LZ/P-like *sycp1*^*isa/isa*^ spermatocytes (Figures 6Avi, Civ). We cannot eliminate the possibility that DSBs are continuously formed at these sites with Dmc1/Rad51 and RPA foci at LZ/P-like stages. However, given that Iho1 was mostly absent from these sites in the LZ/P-like stages (Figure 8Avi), this is less likely to be the case. Collectively, our data support the idea that Sycp1 is essential for homolog association via crossovers in zebrafish spermatocytes.

### Meiotic DSB Regulations in Zebrafish Spermatocytes

In most species studied so far, meiotic DSBs are directed to distinct regions of the genome, called hotspots (Baudat et al., 2013). In this study, we demonstrated that Iho1 appears on axes at L/EZ stages around the telomere bouquet (Figures 8Ai, Bi), where DSBs predominantly occur, in zebrafish spermatocytes (Figure 6A; Blokhina et al., 2019; Takemoto et al., 2020). The initial loading of Iho1 most likely occurs prior to DSB formation, since Iho1 foci were observed in *spo11*^–/–^ spermatocytes (Figure 8Biii, iv). This implies that these regions harbor properties to recruit DSB-promoting proteins through unknown mechanisms and become hotspots. IHO1 was first identified as a HORMAD1-interacting protein that promotes DSBs in mice (Stanzione et al., 2016). Although axis localization of IHO1 is largely lost in *Hormad1* mutant mice, a recent study indicated that HORMAD1 is not the sole factor required for axis recruitment of IHO1 (Fujiwara et al., 2020). Furthermore, another mouse study reported that DSB formation at the pseudoautosomal region and several chromosome ends are associated with a particular type of minisatellite repeat and HORMAD1-independent accumulation of DSB-promoting proteins, such as REC114, MEI4, IHO1 and MEI1 (Acquaviva et al., 2020). In zebrafish, it remains to be elucidated whether Hormad1 is required for Iho1 localization on axes. It will be of future interest to understand how DSB-promoting proteins are recruited to subtelomeric hotspots in zebrafish.

We demonstrated that the spatiotemporal localizations of Iho1 and Hormad1 are differentially affected by depletion of Sycp1 (Figures 7, 8). This observation supports the idea that dissociation of Hormad1 and Iho1 from axes are regulated by different mechanisms. We observed Hormad1 localization on axes upon and after pairing in *sycp1* mutant spermatocytes (Figure 7Avi), suggesting that SC formation is required for the dissociation of Hormad1 from axes. This is consistent with observations in mice that HORMAD1 is enriched on unsynapsed axes in *Syce1* mutant spermatocytes (Wojtasz et al., 2009). In contrast to Hormad1, intense Iho1 foci mostly disappeared from axes in LZ/P-like *sycp1* mutant spermatocytes (Figure 8Avi), and subtelomeric DSBs were observed (Figure 6, Supplementary Figure 4). In addition, persistent Iho1 foci were observed in *spo11*^–/–^ spermatocytes (Figure 8iv). Hence, the dissociation of Iho1 from axes seems to be promoted by DSB formation but not by synapsis. A recent report in mice showed that DSB-dependent IHO1 depletion is promoted locally in the vicinity of DSBs by the ATR kinase at pre-pachynema, and globally in all axes by the DNA damage response signaling pathway at pachynema (Dereli et al., 2021). It is presumable that Iho1 dissociation in *sycp1*^–/–^ zebrafish occurs in similar pathways activated by DSBs, but all-axis depletion of Iho1 might occur prior to the pachytene stage, shortly after DSB formation around the telomere bouquet. Though further studies are required to unveil the complete picture of DSB regulation, our study provided the first insight into the feedback control of DSB protein in zebrafish.

## Materials and Methods

### Ethics Statement

All experiments were conducted in accordance with Rules for Animal Experiments at the National Institute of Genetics, Research Organization of Information and Systems, Japan. The experimental plan using zebrafish was approved by the National Institute of Genetics official ethics committee (Approval Number 27–12, 28–13, 29–13, 30–14, 31–18, R2-8).

### Fish

Zebrafish (*Danio rerio*) were maintained under standard conditions as described in The Zebrafish Book (Westerfield, 1995). The wild-type India line (see ZFIN at http://zfin.org/action/genotype/view/ZDB-GENO-980210-28) has been maintained in our laboratory for more than 20 generations. The *isa* fish have been described previously: *isa* mutant fish were derived from the wild-type Tübingen line and then crossed with the IM strain (wild-type inbred) (Shinya and Sakai, 2011) to obtain heterozygous mutant fish (Saito et al., 2011). The *sycp1^*del*5^* line was generated by CRISPR-Cas9 mutagenesis based on published protocols (Hwang et al., 2013; Chen et al., 2017). Template DNA for single-guide RNA (sgRNA) synthesis was prepared by amplification with a primer specific to *sycp1* exon 3, a universal reverse primer (Supplementary Table 1) and T4 DNA polymerase. After purification of the template DNA, sgRNA was transcribed *in vitro* with a MEGAscript T7 kit (Ambion) and purified with a MEGA clean-up kit (Ambion). Wild-type India embryos were injected at the 1- or 2-cell stage with 2.3 nl of a mixture of 10 pmol/μl Cas9 NLS protein (abm) and 100 ng/μl *sycp1* sgRNA. Founders were backcrossed with India fish, and the F1 siblings were screened by genotyping. Heterozygous *sycp1* knockout fish carrying a −5 bp frameshift mutation in exon 3 (*sycp1^*del*5^*, a 5-bp deletion affecting the codons for Leu78 and Pro79 that generates 10 amino acid residues from the wrong frame and stop codon after the 87th amino acid) were mated with heterozygous *isa* fish in complementation tests.

### Mapping of the *isa* Mutation

We first analyzed 560 *isa* mutant fish with simple sequence polymorphism (SSLP) markers, and the causal genomic region was mapped to a 1197-kbp region on chromosome 6 containing 22 annotated genes. We further analyzed 676 (total 1236) *isa* mutant fish and narrowed it down to an ∼561-kbp region between the A4891 and z4950 markers (Supplementary Figure 1). Among 14 genes located in this region, we examined the genomic sequences of *tshb*, *vapb*, and *sycp1* genes and identified no mutation in *tshb* and *vapb* but a premature stop mutation in *sycp1*.

### Genotyping

Genomic DNA was extracted from caudal fin clips (adult) or head (juvenile) tissue in lysis buffer at 65°C. DNA lysates were diluted 120 times in water and used for PCR with GoTaq Green Master Mix (Promega) using specific primers for each site (Supplementary Table 1). PCR products were examined by agarose or acrylamide gel electrophoresis. Genotyping of the *isa* mutation was performed by the dCAPS (derived cleaved amplified polymorphic sequences) method using *Kpn*I (Nippon Gene). For genotyping of *sycp1^*del*5^* and the *spo11* mutation, PCR products were treated with ExoSAP-IT PCR Product Cleanup Reagent (Thermo Fisher Scientific) and analyzed by general Sanger sequencing.

### Antibodies

All antibodies used in this study are listed in Supplementary Table 2. Our anti-Dmc1/Rad51 antibody was raised against recombinant Dmc1 protein, which shares high similarity with Rad51 at the amino acid sequence level, and thus the antibody potentially recognize Rad51 as well (Takemoto et al., 2020). Mouse polyclonal antibodies specific for zebrafish Hormad1 and Iho1 were generated in this study (Supplementary Figure 6). The *hormad1* and *iho1* cDNAs encoding amino acid residues Ser252 to Lys356 (UniProt ID: A2BF66) and Met1 to Pro306 (NCBI Reference: XP_021332557.1), respectively, were cloned into the pET-28a(+) vector (Novagen) using NEBuilder HiFi DNA Assembly (NEB). Primers used for cloning are shown in Supplementary Table 1. The cloned zebrafish Hormad1 region corresponds to a C-terminal region that was used for the generation of an anti-mouse HORMAD1 antibody to avoid cross-reaction with the HORMAD2 protein (Wojtasz et al., 2009). The recombinant Hormad1 and Iho1 proteins were expressed in *Escherichia coli* BL21C and purified by Ni-NTA agarose (Qiagen) under denaturing conditions (6 M HCl-guanidine, 20 mM Tris–HCl pH 7.5). After immunizing mice with the recombinant proteins, the obtained anti-Hormad1 antiserum was purified by Dynabeads protein G.

### Western Blotting

Protein extracts were prepared from testes dissected from 3.5-month-old fish. A pair of testes was homogenized in 100 μl of extraction buffer (20 mM Tris; 150 mM NaCl; 2 mM EDTA; 1% NP-40; 0.1% SDS) complemented with cOmplete proteinase inhibitors (Roche). After sonication by a Bioruptor at high power for 5 cycles of 30 s on/30 s off, cell suspensions were incubated at 4°C for 30 minutes. Protein lysates were obtained by centrifugation at 14,500 rpm for 10 min at 4°C. Protein concentrations were determined by Bio-Rad Protein Assay (Bio-Rad), and 25 μg protein was loaded onto a 7.5% SuperSep(TM) Ace acrylamide gel (Wako) and migrated at 200 V for 75 min. The proteins were transferred onto a PVDF membrane by the Trans-Blot Turbo system (Bio-Rad), and then the membrane was blocked in 5% skim milk and TBST. The membrane was cut at ∼40 kDa as indicated by the position of protein markers and then incubated with anti-Sycp1 or anti-Gapdh antibodies. After washing 3 times in TBST, the membrane was blotted with HRP-conjugated secondary antibodies. The antibodies used in western blotting are listed in Supplementary Table 2. Signals were developed with ECL Prime reagent (Amersham) and detected by ChemiDoc XRS (Bio-Rad).

### Histology

Adult testes and bodies of juvenile fish (24 to 45 dpf) were fixed in Bouin’s solution (Sigma). For sampling of juvenile fish, total body size (from the snout to the end of the caudal fin) was measured, and either the fin clip or the head was kept for genotyping. The fixed samples were dehydrated in an ethanol series (70%, 80%, 90% and 100%), methyl benzoate, and Lemosol (Wako) and embedded in paraffin. Hematoxylin-eosin (HE) staining was performed with 5-μm-thick sections.

### Immunostaining of Spermatocyte Chromosome Spreads

Zebrafish spermatocyte chromosome spreads were prepared from adult testes by a dry-down method as described previously (Takemoto et al., 2020). Blocking of the spread slides was performed with a solution of PBS containing 5% skimmed milk and 5% donkey serum. The slides were incubated at room temperature (RT) overnight with primary antibodies and at 37°C for 1 h with secondary antibodies. All antibodies and their dilutions at the time of use are listed in Supplementary Table 2. After each antibody incubation, slides were washed for 10 min each in 0.1% Tween 20-PBS, 0.1% Triton X-100-PBS, and 0.1% Tween 20-PBS. DAPI was added at 10 ng/ml in the last wash step. After washing the slides twice in distilled water, they were air dried and mounted using VECTASHIELD Antifade Mounting Medium (Vector Labs). For telomere staining, TEN buffer (100 mM NaCl, 20 mM Tris–HCl pH 7.4, 1 mM EDTA) or TE buffer (20 mM Tris–HCl pH 7.4, 1 mM EDTA, for the wash step before incubation with the secondary antibody mix) was used instead of PBS, and incubation with secondary antibodies was performed with 0.5 μg/ml DAPI and 15 nM TRed-HPTH59-A (telomere-targeting polyamide; HiPep Laboratories) (Sasaki et al., 2016) at RT for 3–4 h.

### DNA FISH

The fosmid probe and Cot-1 DNAs were prepared as previously described using the fosmid clone CH1073-175H21 (BACPAC Genomics) and salmon sperm DNA (Wako), respectively (Blokhina et al., 2019). We confirmed that CH1073-175H21 contains a genomic region close to a telomere on chromosome 3 by PCR with a primer pair specific to the *sox9b* gene (Supplementary Table 1). The DNA probe staining protocol was adapted from Takemoto et al. (2020). After immunostaining of the chromosome spreads as described above, the slides were postfixed with 500 μl of 2% paraformaldehyde in PBS for 10 min at RT. The slides were then washed in PBS and dehydrated by placing in 70%, 85% and (twice) 100% ethanol. Dried slides were incubated with 10 μl of probe mixed with 25 μg of Cot-1 at 80°C for 5 min. After coming to temperature at ∼37°C, slides were incubated in a humid chamber at 37°C overnight. Slides were then washed twice in 50% formamide in 2xSSC at 45°C for 5 min, twice in 2x SSC at 45°C for 5 min, once in 0.05% Tween20-4xSSC for at RT 8 min, once in 0.05% Tween20-1xSSC-0.5xPBS at RT for 5 min, and finally 3 times in 0.1% Tween 20-PBS at RT for 5 min. The slides were then stained with DAPI and mounted as described above.

### Image Analysis

Histological images were captured with an Olympus BX51 microscope equipped with a Keyence VB7010 camera. Cytological images were captured with a DeltaVision Ultra fluorescence microscope equipped with a 1.45 NA 100x objective, and 20 images along the z-axis (section spacing: 0.1 μm) were deconvolved and projected using softWoRx software (GE Healthcare). All cytological images were processed using OMERO (OME) (Allan et al., 2012) and Fiji (Schindelin et al., 2012). All spread samples included in the same panel were prepared and stained at the same time, and the images were processed in the same manner. Quantifications were performed by Fiji macros using DAPI-positive areas as ROIs (programs available upon request). The same threshold was applied to all images in the same experiment to eliminate noise or background signals. The numbers of telomeres (TPA; Figure 4B) and Dmc1/Rad51 foci (Figure 6B) were counted by the FindMaxima tool in Fiji. The distance between two FISH foci was quantified as previously described (Blokhina et al., 2019). When only one FISH focus was detected in the nucleus, the radius of the FISH-stained area was considered to indicate the distance between two FISH foci. To quantify Dmc1/Rad51 foci in near telomeres, we defined telomere-proximal regions as the 1 μm of axis adjacent to the TPA focus, based on a criterion used for human DMC1 analysis (Pratto et al., 2014). We manually counted numbers of Dmc1/Rad51 foci in telomere-proximal regions in all MZ and MZ-like nuclei quantified in Figure 6B, and calculated proportion of Dmc1/Rad51 foci in telomere-proximal regions to total Dmc1/Rad51 foci in each nucleus.

### Statistics

Two or more animals (or siblings in Figure 2) were analyzed as biological replicates for the same phenotypes, unless otherwise indicated. Each phenotype was compared between wild-type and mutant animals from the same brood (siblings). The significance of the numbers of TPA, Dmc1/Rad51 and RPA foci and intensities of γH2AX and RPA signals was calculated with the Mann–Whitney two-tailed test using GraphPad Prism 9 software.

## Data Availability Statement

The data generated for this study can be found within the article/Supplementary Material.

## Ethics Statement

The animal study was reviewed and approved by Animal Experiments at the National Institute of Genetics, Research Organization of Information and Systems.

## Author Contributions

YI contributed to the conceptualization, investigation, formal analysis, visualization, writing of the original draft, and funding acquisition. KS contributed to the conceptualization, investigation, and formal analysis. KT and FV contributed to the investigation and formal analysis. TK contributed to project supervision. KI contributed to project supervision and resources for the project. NS contributed to the conceptualization, supervision, project administration, funding acquisition, and resources for the project. All authors revised, commented on, and approved the final manuscript.

## Conflict of Interest

The authors declare that the research was conducted in the absence of any commercial or financial relationships that could be construed as a potential conflict of interest.

## Supplementary Material

Supplementary Figure 1 Mapping of the *isa* mutation with SSLP markers. The names of the SSLP markers used and their locations on chromosome 6 (in Mb, the Zv9 zebrafish reference genome) are shown with mapping results. Tu: Tubingen alleles associated with the ENU-mutated line)/IM: inbred India alleles associated with the wild-type line used for backcrossing. Sixteen genes found in an ∼561-kbp region between markers A4891 and z4950 are listed below.

Supplementary Figure 2 HE-stained sections of *sycp1*^+/+^, *sycp1*^*isa/isa*^ and *sycp1^*del*5/del5^* testes. **(A)** images of *sycp1*^+/+^ and *sycp1*^*isa/isa*^ testes from siblings at 4 mpf. **(B)** images of *sycp1*^+/+^ and *sycp1^*del*5/del5^* testes from siblings at 4 mpf. Representative results of two individual fish are shown for each genotype. Sc: spermatocytes, St: spermatids, Sz: spermatozoa. Lumens with no spermatozoa (asterisks) and spermatocytes with irregular nuclei (inside broken lines) were observed in the *sycp1*^*isa/isa*^ section, as previously reported (Saito et al., 2014), as well as in the *sycp1^*del*5/del5^* section.

Supplementary Figure 3 HE-stained sections of juvenile gonads. Images of *sycp1*^+/+^ **(A)** and *sycp1*^*isa/isa*^ **(B)** gonads are shown with fish ID, size (mm) and age (dpf). Samples were obtained from two different siblings indicated in red and black letters. The largest oocytes found on each section of gonads with oocytes are indicated with arrows. For gonads with spermatocysts, examples of spermatocysts are marked with white broken lines. Note that both oocytes and spermatocysts were observed in wt29 gonads (both images are shown here) and that neither oocytes nor spermatocysts were observed in mut14 gonads. These two fish were categorized as N.D. (not determined) in Figure 2C. Scale bars, 20 μm.

Supplementary Figure 4 Proportion of Dmc1/Rad51 foci in telomere-proximal regions. The number of Dmc1/Rad51 foci in telomere-proximal regions was counted in the MZ and MZ-like nuclei quantified for total Dmc1/Rad51 foci in Figure 6B. Proportion of Dmc1/Rad51 foci in telomere-proximal regions to total Dmc1/Rad51 foci in a nucleus was plotted for MZ *sycp1*^+/+^ and MZ-like *sycp1*^*isa/isa*^ spermatocytes.

Supplementary Figure 5 Quantification of RPA foci in *sycp1*^+/+^ and *sycp1*^*isa/isa*^ spermatocytes. The number of RPA foci was counted in the same nuclei quantified for RPA signal intensities, as shown in Figure 6D. Black bars indicate means. Statistical significance was examined by a two-tailed Mann-Whitney test (^∗^*P* < 0.1, ^∗∗∗^*P* < 0.001, ns, not significant; exact *P* value).

Supplementary Figure 6 Generation of antibodies against zebrafish Hormad1 and Iho1. **(A)** Western blotting of wild-type testis protein extracts with anti-zebrafish Hormad1 antisera obtained from five immunized mice (M1 to M5), unimmunized mouse serum (Ctrl) and no-serum control (2nd Ab only). The blot image is superposed with a binary image of colorimetric capture of molecular ladders (in blue). The band indicated with a red arrowhead is close to the predicted size of the full-length Hormad1 protein (41 kDa). Each well was loaded with 50 μg protein. M1 serum was used for immunostaining in this study (red). **(B)** Western blotting of protein extracts from *Iho1*^+/+^ and *Iho1*^–/–^ testes with anti-zebrafish Iho1 antisera obtained from immunized mice. The predicted size of the full-length Iho1 protein is 61 kDa, and the band specific to the *Iho1*^+/+^ sample is indicated with a red arrow. Each well was loaded with 50 μg protein. **(C)** Coimmunostaining of Iho1 and Sycp3 on *Iho1*^+/+^ and *Iho1*^–/–^ spermatocyte spreads. Iho1 signals were barely observed in *Iho1*^–/–^ spermatocytes.

Supplementary Table 1 List of primers used in this study.

Supplementary Table 2 List of antibodies used in this study.

## References

[B1] Acquaviva L. Boekhout M. Karasu M. E. Brick K. Pratto F. Li T. (2020). Ensuring meiotic DNA break formation in the mouse pseudoautosomal region. *Nature* 582 426–431. 10.1038/s41586-020-2327-4 32461690 PMC7337327

[B2] Allan C. Burel J.-M. Moore J. Blackburn C. Linkert M. Loynton S. (2012). OMERO: flexible, model-driven data management for experimental biology. *Nat. Methods* 9 245–253. 10.1038/nmeth.1896 22373911 PMC3437820

[B3] Baker K. E. Parker R. (2004). Nonsense-mediated mRNA decay: terminating erroneous gene expression. *Curr. Opin. Cell Biol.* 16 293–299. 10.1016/j.ceb.2004.03.003 15145354

[B4] Barlow A. L. Benson F. E. West S. C. Hultén M. A. (1997). Distribution of the Rad51 recombinase in human and mouse spermatocytes. *EMBO J.* 16 5207–5215. 10.1093/emboj/16.17.5207 9311981 PMC1170153

[B5] Barlow A. L. Hultén M. A. (1998). Crossing over analysis at pachytene in man. *Eur. J. Hum. Genet.* 6 350–358. 10.1038/sj.ejhg.5200200 9781043

[B6] Baudat F. Imai Y. de Massy B. (2013). Meiotic recombination in mammals: localization and regulation. *Nat. Rev. Genet.* 14 794–806. 10.1038/nrg3573 24136506

[B7] Bergerat A. de Massy B. Gadelle D. Varoutas P.-C. Nicolas A. Forterre P. (1997). An atypical topoisomerase II from archaea with implications for meiotic recombination. *Nature* 386 414–417. 10.1038/386414a0 9121560

[B8] Bisig C. G. Guiraldelli M. F. Kouznetsova A. Scherthan H. Höög C. Dawson D. S. (2012). Synaptonemal complex components persist at centromeres and are required for homologous centromere pairing in mouse spermatocytes. *PLoS Genet.* 8:e1002701. 10.1371/journal.pgen.1002701 22761579 PMC3386160

[B9] Blokhina Y. P. Nguyen A. D. Draper B. W. Burgess S. M. (2019). The telomere bouquet is a hub where meiotic double-strand breaks, synapsis, and stable homolog juxtaposition are coordinated in the zebrafish, *Danio rerio*. *PLoS Genet.* 15:e1007730. 10.1371/journal.pgen.1007730 30653507 PMC6336226

[B10] Boekhout M. Karasu M. E. Wang J. Acquaviva L. Pratto F. Brick K. (2019). REC114 partner ANKRD31 controls number, timing and location of meiotic DNA breaks. *Mol. Cell* 74 1053–1068.e8. 10.1016/j.molcel.2019.03.023. 31003867 PMC6555648

[B11] Bolcun-Filas E. Costa Y. Speed R. Taggart M. Benavente R. De Rooij D. G. (2007). SYCE2 is required for synaptonemal complex assembly, double strand break repair, and homologous recombination. *J. Cell Biol.* 176 741–747. 10.1083/jcb.200610027 17339376 PMC2064047

[B12] Bolcun-Filas E. Schimenti J. C. (2012). Genetics of meiosis and recombination in mice. *Int. Rev. Cell Mol. Biol.* 298 179–227. 10.1016/B978-0-12-394309-5.00005-5 22878107

[B13] Bolcun-Filas E. Hall E. Speed R. Taggart M. Grey C. de Massy B. (2009). Mutation of the mouse syce1 gene disrupts synapsis and suggests a link between synaptonemal complex structural components and DNA repair. *PLoS Genet.* 5:e1000393. 10.1371/journal.pgen.1000393 19247432 PMC2640461

[B14] Brick K. Smagulova F. Khil P. Camerini-Otero R. D. Petukhova G. V. (2012). Genetic recombination is directed away from functional genomic elements in mice. *Nature* 485 642–645. 10.1038/nature11089 22660327 PMC3367396

[B15] Brick K. Thibault-Sennett S. Smagulova F. Lam K.-W. G. Pu Y. Pratto F. (2018). Extensive sex differences at the initiation of genetic recombination. *Nature* 561 338–342. 10.1038/s41586-018-0492-5 30185906 PMC6364566

[B16] Brown M. S. Bishop D. K. (2015). DNA strand exchange and RecA homologs in meiosis. *Cold Spring Harb. Perspect. Biol.* 7:a016659. 10.1101/cshperspect.a016659 25475089 PMC4292170

[B17] Brown P. W. Judis L. Chan E. R. Schwartz S. Seftel A. Thomas A. (2005). Meiotic synapsis proceeds from a limited number of subtelomeric sites in the human male. *Am. J. Hum. Genet.* 77 556–566. 10.1086/468188 16175502 PMC1275605

[B18] Chen Y. Zeng S. Hu R. Wang X. Huang W. Liu J. (2017). Using local chromatin structure to improve CRISPR/Cas9 efficiency in zebrafish. *PLoS One* 12:e0182528. 10.1371/journal.pone.0182528 28800611 PMC5553855

[B19] Coop G. Wen X. Ober C. Pritchard J. K. Przeworski M. (2008). High-resolution mapping of crossovers reveals extensive variation in fine-scale recombination patterns among humans. *Science* 319 1395–1398. 10.1126/science.1151851 18239090

[B20] Costa Y. Speed R. Öllinger R. Alsheimer M. Semple C. A. Gautier P. (2005). Two novel proteins recruited by synaptonemal complex protein 1 (SYCP1) are at the centre of meiosis. *J. Cell Sci.* 118 2755–2762. 10.1242/jcs.02402 15944401

[B21] de Massy B. (2013). Initiation of meiotic recombination: how and where? conservation and specificities among eukaryotes. *Ann. Rev. Genet.* 47 563–599. 10.1146/annurev-genet-110711-155423 24050176

[B22] de Vries F. A. T. de Boer E. van den Bosch M. Baarends WM. Ooms M. Yuan L. (2005). Mouse Sycp1 functions in synaptonemal complex assembly, meiotic recombination, and XY body formation. *Genes Dev.* 19 1376–1389. 10.1101/gad.329705 15937223 PMC1142560

[B23] de Vries L. Behar D. M. Smirin-Yosef P. Lagovsky I. Tzur S. Basel-Vanagaite L. (2014). Exome sequencing reveals SYCE1 mutation associated with autosomal recessive primary ovarian insufficiency. *J. Clin. Endocrinol. Metab.* 99 E2129–E2132. 10.1210/jc.2014-1268 25062452

[B24] Dereli I. Stanzione M. Olmeda F. Papanikos F. Baumann M. Demir S. (2021). pronged Four- negative feedback of DSB machinery in meiotic DNA-break control in mice. *Nucleic Acids Res.* 10.1093/nar/gkab082 [Epub ahead of print]. 33619545 PMC7969012

[B25] Draper B. W. McCallum C. M. Moens C. B. (2007). nanos1 is required to maintain oocyte production in adult zebrafish. *Dev. Biol.* 305 589–598. 10.1016/j.ydbio.2007.03.007 17418113 PMC1986726

[B26] Dunce J. M. Dunne O. M. Ratcliff M. Millán C. Madgwick S. Usón I. (2018). Structural basis of meiotic chromosome synapsis through SYCP1 self-assembly. *Nat. Struct. Mol. Biol.* 25 557–569. 10.1038/s41594-018-0078-9 29915389 PMC6606445

[B27] Elkouby Y. M. Mullins M. C. (2017). Methods for the analysis of early oogenesis in zebrafish. *Dev. Biol.* 430 310–324. 10.1016/j.ydbio.2016.12.014 27988227 PMC5555829

[B28] Feitsma H. Leal M. C. Moens P. B. Cuppen E. Schulz R. W. (2007). Mlh1 deficiency in zebrafish results in male sterility and aneuploid as well as triploid progeny in females. *Genetics* 175 1561–1569. 10.1534/genetics.106.068171 17237513 PMC1855116

[B29] Fraune J. Alsheimer M. Volff J.-N. Busch K. Fraune S. Bosch T. C. G. (2012). Hydra meiosis reveals unexpected conservation of structural synaptonemal complex proteins across metazoans. *Proc. Natl. Acad. Sci. U.S.A.* 109 16588–16593. 10.1073/pnas.1206875109 23012415 PMC3478637

[B30] Fujiwara Y. Horisawa-Takada Y. Inoue E. Tani N. Shibuya H. Fujimura S. (2020). Meiotic cohesins mediate initial loading of HORMAD1 to the chromosomes and coordinate SC formation during meiotic prophase. *PLoS Genet.* 16:e1009048. 10.1371/journal.pgen.1009048 32931493 PMC7518614

[B31] Fukuda T. Daniel K. Wojtasz L. Toth A. Höög C. (2010). A novel mammalian HORMA domain-containing protein, HORMAD1, preferentially associates with unsynapsed meiotic chromosomes. *Exp. Cell Res.* 316 158–171. 10.1016/j.yexcr.2009.08.007 19686734

[B32] Gautier A. Goupil A.-S. Le Gac F. Lareyre J.-J. (2013). A promoter fragment of the sycp1 gene is sufficient to drive transgene expression in male and female meiotic germ cells in zebrafish. *Biol. Reprod.* 89 1–14. 10.1095/biolreprod.113.107706 23966324

[B33] Gómez-H L. Felipe-Medina N. Sánchez-Martín M. Davies O. R. Ramos I. García-Tuñón I. (2016). C14ORF39/SIX6OS1 is a constituent of the synaptonemal complex and is essential for mouse fertility. *Nat. Commun.* 7:13298. 10.1038/ncomms13298 27796301 PMC5095591

[B34] Gray S. Cohen P. E. (2016). Control of meiotic crossovers: from double-strand break formation to designation. *Annu. Rev. Genet.* 50 175–210. 10.1146/annurev-genet-120215-035111 27648641 PMC5319444

[B35] Grishaeva T. M. Bogdanov Y. F. (2014). Conservation and variability of synaptonemal complex proteins in phylogenesis of eukaryotes. *Int. J. Evol. Biol.* 2014:856230. 10.1155/2014/856230 25147749 PMC4132317

[B36] Gruhn J. R. Al-Asmar N. Fasnacht R. Maylor-Hagen H. Peinado V. Rubio C. (2016). Correlations between synaptic initiation and meiotic recombination: a study of humans and Mice. *Am. J. Hum. Genet.* 98 102–115. 10.1016/j.ajhg.2015.11.019 26749305 PMC4716685

[B37] Hamer G. Gell K. Kouznetsova A. Novak I. Benavente R. Höög C. (2006). Characterization of a novel meiosis-specific protein within the central element of the synaptonemal complex. *J. Cell Sci.* 119 4025–4032. 10.1242/jcs.03182 16968740

[B38] Hamer G. Wang H. Bolcun-Filas E. Cooke H. J. Benavente R. Höög C. (2008). Progression of meiotic recombination requires structural maturation of the central element of the synaptonemal complex. *J. Cell Sci.* 121 2445–2451. 10.1242/jcs.033233 18611960

[B39] Higgins J. D. Sanchez-Moran E. Armstrong S. J. Jones G. H. Franklin F. C. H. (2005). The *Arabidopsis* synaptonemal complex protein ZYP1 is required for chromosome synapsis and normal fidelity of crossing over. *Genes Dev.* 19 2488–2500. 10.1101/gad.354705 16230536 PMC1257403

[B40] Hinch A. G. Becker P. W. Li T. Moralli D. Zhang G. Bycroft C. (2020). The configuration of RPA, RAD51, and DMC1 binding in meiosis reveals the nature of critical recombination intermediates. *Mol. Cell* 79 689–701.e10. 10.1016/j.molcel.2020.06.015 32610038 PMC7447979

[B41] Houwing S. Kamminga L. M. Berezikov E. Cronembold D. Girard A. van den Elst H. (2007). A role for Piwi and piRNAs in germ cell maintenance and transposon silencing in zebrafish. *Cell* 129 69–82. 10.1016/j.cell.2007.03.026 17418787

[B42] Hunter N. (2015). Meiotic recombination: the essence of heredity. *Cold Spring Harb. Perspect. Biol.* 7:a016618. 10.1101/cshperspect.a016618 26511629 PMC4665078

[B43] Hwang W. Y. Fu Y. Reyon D. Maeder M. L. Tsai S. Q. Sander J. D. (2013). Efficient genome editing in zebrafish using a CRISPR-Cas system. *Nat. Biotech.* 31 227–229. 10.1038/nbt.2501 23360964 PMC3686313

[B44] Kamminga L. M. Luteijn M. J. den Broeder M. J. Redl S. Kaaij L. J. T. Roovers E. K. (2010). Hen1 is required for oocyte development and piRNA stability in zebrafish. *EMBO J.* 29 3688–3700. 10.1038/emboj.2010.233 20859253 PMC2982757

[B45] Keeney S. Giroux C. N. Kleckner N. (1997). Meiosis-specific DNA double-strand breaks are catalyzed by Spo11, a member of a widely conserved protein family. *Cell* 88 375–384. 10.1016/S0092-8674(00)81876-0 9039264

[B46] Khil P. P. Camerini-Otero R. D. (2010). Genetic crossovers are predicted accurately by the computed human recombination map. *PLoS Genet.* 6:e1000831. 10.1371/journal.pgen.1000831 20126534 PMC2813264

[B47] Kirkness E. F. Grindberg R. V. Yee-Greenbaum J. Marshall C. R. Scherer S. W. Lasken R. S. (2013). Sequencing of isolated sperm cells for direct haplotyping of a human genome. *Genome Res.* 23 826–832. 10.1101/gr.144600.112 23282328 PMC3638138

[B48] Kochakpour N. Moens P. B. (2008). Sex-specific crossover patterns in Zebrafish (*Danio rerio*). *Heredity* 100 489–495. 10.1038/sj.hdy.6801091 18322458

[B49] Kong A. Gudbjartsson D. F. Sainz J. Jonsdottir G. M. Gudjonsson S. A. Richardsson B. (2002). A high-resolution recombination map of the human genome. *Nat. Genet.* 31 241–247. 10.1038/ng917 12053178

[B50] Kumar R. Bourbon H.-M. de Massy B. (2010). Functional conservation of Mei4 for meiotic DNA double-strand break formation from yeasts to mice. *Genes Dev.* 24 1266–1280. 10.1101/gad.571710 20551173 PMC2885662

[B51] Kumar R. Oliver C. Brun C. Juarez-Martinez A. B. Tarabay Y. Kadlec J. (2018). Mouse REC114 is essential for meiotic DNA double-strand break formation and forms a complex with MEI4. *Life Sci. Alliance* 1:e201800259. 10.26508/lsa.201800259 30569039 PMC6288613

[B52] Lewis B. P. Green R. E. Brenner S. E. (2003). Evidence for the widespread coupling of alternative splicing and nonsense-mediated mRNA decay in humans. *Proc. Natl. Acad. Sci. U.S.A.* 100 189–192. 10.1073/pnas.0136770100 12502788 PMC140922

[B53] Liew W. C. Bartfai R. Lim Z. Sreenivasan R. Siegfried K. R. Orban L. (2012). Polygenic sex determination system in zebrafish. *PLoS One* 7:e34397. 10.1371/journal.pone.0034397 22506019 PMC3323597

[B54] Lu S. Zong C. Fan W. Yang M. Li J. Chapman A. R. (2012). Probing meiotic recombination and aneuploidy of single sperm cells by whole genome sequencing. *Science* 338 1627–1630. 10.1126/science.1229112 23258895 PMC3590491

[B55] Maack G. Segner H. (2003). Morphological development of the gonads in zebrafish. *J. Fish Biol.* 62 895–906. 10.1046/j.1095-8649.2003.00074.x

[B56] MacQueen A. J. Colaiácovo M. P. McDonald K. Villeneuve A. M. (2002). Synapsis-dependent and -independent mechanisms stabilize homolog pairing during meiotic prophase in C. elegans. *Genes Dev.* 16 2428–2442. 10.1101/gad.1011602 12231631 PMC187442

[B57] Mahadevaiah S. K. Turner J. M. A. Baudat F. Rogakou E. P. de Boer P. Blanco-Rodríguez J. (2001). Recombinational DNA double-strand breaks in mice precede synapsis. *Nat. Genet.* 27 271–276. 10.1038/85830 11242108

[B58] Malik S.-B. Ramesh M. A. Hulstrand A. M. Logsdon J. M. (2007). Protist homologs of the meiotic Spo11 gene and topoisomerase VI reveal an evolutionary history of gene duplication and lineage-specific loss. *Mol. Biol. Evol.* 24 2827–2841. 10.1093/molbev/msm217 17921483

[B59] Meuwissen R. L. Offenberg H. H. Dietrich A. J. Riesewijk A. van Iersel M. Heyting C. (1992). A coiled-coil related protein specific for synapsed regions of meiotic prophase chromosomes. *EMBO J.* 11 5091–5100. 10.1002/j.1460-2075.1992.tb05616.x 1464329 PMC556987

[B60] Moens P. B. (2006). Zebrafish: chiasmata and interference. *Genome* 49 205–208. 10.1139/g06-021 16604102

[B61] Osman K. Sanchez-Moran E. Higgins J. D. Jones G. H. Franklin F. C. H. (2006). Chromosome synapsis in *Arabidopsis*: analysis of the transverse filament protein ZYP1 reveals novel functions for the synaptonemal complex. *Chromosoma* 115:212. 10.1007/s00412-005-0042-4 16421735

[B62] Papanikos F. Clément J. A. J. Testa E. Ravindranathan R. Grey C. Dereli I. (2019). Mouse ANKRD31 regulates spatiotemporal patterning of meiotic recombination initiation and ensures recombination between X and Y Sex chromosomes. *Mol. Cell* 74 1069–1085.e11. 10.1016/j.molcel.2019.03.022 31000436

[B63] Pratto F. Brick K. Khil P. Smagulova F. Petukhova G. V. Camerini-Otero R. D. (2014). Recombination initiation maps of individual human genomes. *Science* 346 1256442. 10.1126/science.1256442 25395542 PMC5588152

[B64] Robert T. Nore A. Brun C. Maffre C. Crimi B. Guichard V. (2016). The TopoVIB-Like protein family is required for meiotic DNA double-strand break formation. *Science* 351 943–949. 10.1126/science.aad5309 26917764

[B65] Rodríguez-Marí A. Cañestro C. BreMiller R. A. Nguyen-Johnson A. Asakawa K. Kawakami K. (2010). Sex reversal in zebrafish fancl mutants is caused by Tp53-mediated germ cell apoptosis. *PLoS Genet.* 6:e1001034. 10.1371/journal.pgen.1001034 20661450 PMC2908690

[B66] Rodríguez-Marí A. Yan Y.-L. BreMiller R. A. Wilson C. Cañestro C. Postlethwait J. H. (2005). Characterization and expression pattern of zebrafish anti-Müllerian hormone (amh) relative to sox9a, sox9b, and cyp19a1a, during gonad development. *Gene Expr. Patterns* 5 655–667. 10.1016/j.modgep.2005.02.008 15939378

[B67] Saito K. Sakai C. Kawasaki T. Sakai N. (2014). Telomere distribution pattern and synapsis initiation during spermatogenesis in zebrafish. *Dev. Dyn.* 243 1448–1456. 10.1002/dvdy.24166 25044979

[B68] Saito K. Siegfried K. R. Nüsslein-Volhard C. Sakai N. (2011). Isolation and cytogenetic characterization of zebrafish meiotic prophase I mutants. *Dev. Dyn.* 240 1779–1792. 10.1002/dvdy.22661 21594953

[B69] Sansam C. L. Pezza R. J. (2015). Connecting by breaking and repairing: mechanisms of DNA strand exchange in meiotic recombination. *FEBS J.* 282 2444–2457. 10.1111/febs.13317 25953379 PMC4573575

[B70] Sasaki A. Ide S. Kawamoto Y. Bando T. Murata Y. Shimura M. (2016). Telomere visualization in tissue sections using pyrrole–imidazole polyamide probes. *Sci. Rep.* 6:29261. 10.1038/srep29261 27380936 PMC4933941

[B71] Schindelin J. Arganda-Carreras I. Frise E. Kaynig V. Longair M. Pietzsch T. (2012). Fiji: an open-source platform for biological-image analysis. *Nat. Methods* 9 676–682. 10.1038/nmeth.2019 22743772 PMC3855844

[B72] Schramm S. Fraune J. Naumann R. Hernandez-Hernandez A. Höög C. Cooke H. J. (2011). A novel mouse synaptonemal complex protein is essential for loading of central element proteins, recombination, and fertility. *PLoS Genet.* 7:e1002088. 10.1371/journal.pgen.1002088 21637789 PMC3102746

[B73] Scully R. Xie A. (2013). Double strand break repair functions of histone H2AX. *Mutat. Res.* 750 5–14. 10.1016/j.mrfmmm.2013.07.007 23916969 PMC3818383

[B74] Selman K. Wallace R. A. Sarka A. Qi X. (1993). Stages of oocyte development in the zebrafish, *Brachydanio rerio*. *J. Morphol.* 218 203–224. 10.1002/jmor.1052180209 29865471

[B75] Shi B. Xue J. Yin H. Guo R. Luo M. Ye L. (2019). Dual functions for the ssDNA-binding protein RPA in meiotic recombination. *PLoS Genet.* 15:e1007952. 10.1371/journal.pgen.1007952 30716097 PMC6375638

[B76] Shin Y.-H. Choi Y. Erdin S. U. Yatsenko S. A. Kloc M. Yang F. (2010). Hormad1 mutation disrupts synaptonemal complex formation, recombination, and chromosome segregation in mammalian meiosis. *PLoS Genet.* 6:e1001190. 10.1371/journal.pgen.1001190 21079677 PMC2973818

[B77] Shinya M. Sakai N. (2011). Generation of highly homogeneous strains of zebrafish through full sib-pair mating. *G3(Bethesda)* 1 377–386. 10.1534/g3.111.000851 22384348 PMC3276154

[B78] Shive H. R. West R. R. Embree L. J. Azuma M. Sood R. Liu P. (2010). brca2 in zebrafish ovarian development, spermatogenesis, and tumorigenesis. *Proc. Natl. Acad. Sci. U.S.A.* 107 19350–19355. 10.1073/pnas.1011630107 20974951 PMC2984219

[B79] Siegfried K. R. Nüsslein-Volhard C. (2008). Germ line control of female sex determination in zebrafish. *Dev. Biol.* 324 277–287. 10.1016/j.ydbio.2008.09.025 18930041

[B80] Slanchev K. Stebler J. de la Cueva-Méndez G. Raz E. (2005). Development without germ cells: the role of the germ line in zebrafish sex differentiation. *Proc. Natl. Acad. Sci. U.S.A.* 102 4074–4079. 10.1073/pnas.0407475102 15728735 PMC549510

[B81] Smagulova F. Gregoretti I. V. Brick K. Khil P. Camerini-Otero R. D. Petukhova G. V. (2011). Genome-wide analysis reveals novel molecular features of mouse recombination hotspots. *Nature* 472 375–378. 10.1038/nature09869 21460839 PMC3117304

[B82] Soustelle C. Vedel M. Kolodner R. Nicolas A. (2002). Replication protein A is required for meiotic recombination in *Saccharomyces cerevisiae*. *Genetics* 161 535–547. 12072452 10.1093/genetics/161.2.535 PMC1462150

[B83] Stanzione M. Baumann M. Papanikos F. Dereli I. Lange J. Ramlal A. (2016). Meiotic DNA break formation requires the unsynapsed chromosome axis-binding protein IHO1 (CCDC36) in mice. *Nat. Cell Biol.* 18 1208–1220. 10.1038/ncb3417 27723721 PMC5089853

[B84] Sugiyama T. Zaitseva E. M. Kowalczykowski S. C. (1997). A single-stranded DNA-binding protein is needed for efficient presynaptic complex formation by the Saccharomyces cerevisiae Rad51 protein. *J. Biol. Chem.* 272 7940–7945. 10.1074/jbc.272.12.7940 9065463

[B85] Sym M. Engebrecht J. A. Roeder G. S. (1993). ZIP1 is a synaptonemal complex protein required for meiotic chromosome synapsis. *Cell* 72 365–378. 10.1016/0092-8674(93)90114-6 7916652

[B86] Takemoto K. Imai Y. Saito K. Kawasaki T. Carlton P. M. Ishiguro K. (2020). Sycp2 is essential for synaptonemal complex assembly, early meiotic recombination and homologous pairing in zebrafish spermatocytes. *PLoS Genet* 16:e1008640. 10.1371/journal.pgen.1008640 32092049 PMC7062287

[B87] Uchida D. Yamashita M. Kitano T. Iguchi T. (2002). Oocyte apoptosis during the transition from ovary-like tissue to testes during sex differentiation of juvenile zebrafish. *J. Exp. Biol.* 205 711–718. 11914381 10.1242/jeb.205.6.711

[B88] Wang J. Fan H. C. Behr B. Quake S. R. (2012). Genome-wide single-cell analysis of recombination activity and De novo mutation rates in human sperm. *Cell* 150 402–412. 10.1016/j.cell.2012.06.030 22817899 PMC3525523

[B89] Westerfield M. (1995). *The Zebrafish Book. A Guide for the Laboratory Use of Zebrafish (Danio rerio)*, 3rd Edn. Eugene, OR: University of Oregon Press.

[B90] Westergaard M. von Wettstein D. (1972). The synaptinemal complex. *Annu. Rev. Genet.* 6 71–110. 10.1146/annurev.ge.06.120172.000443 4269097

[B91] Wojtasz L. Daniel K. Roig I. Bolcun-Filas E. Xu H. Boonsanay V. (2009). Mouse HORMAD1 and HORMAD2, two conserved meiotic chromosomal proteins, are depleted from synapsed chromosome axes with the help of TRIP13 AAA-ATPase. *PLoS Genet.* 5:e1000702. 10.1371/journal.pgen.1000702 19851446 PMC2758600

[B92] Zickler D. Kleckner N. (1999). Meiotic chromosomes: integrating structure and function. *Annu. Rev. Genet.* 33 603–754. 10.1146/annurev.genet.33.1.603 10690419

[B93] Zickler D. Kleckner N. (2015). Recombination, pairing, and synapsis of homologs during meiosis. *Cold Spring Harb. Perspect. Biol.* 7:a016626. 10.1101/cshperspect.a016626 25986558 PMC4448610

