# Supplementary figures and images for: Sycp1 Is Not Required for Subtelomeric DNA Double-Strand Breaks but Is Required for Homologous Alignment in Zebrafish Spermatocytes
Source: Front Cell Dev Biol. 2021 Mar 26;9:664377. doi: 10.3389/fcell.2021.664377 (PMC8033029; doi:10.3389/fcell.2021.664377)

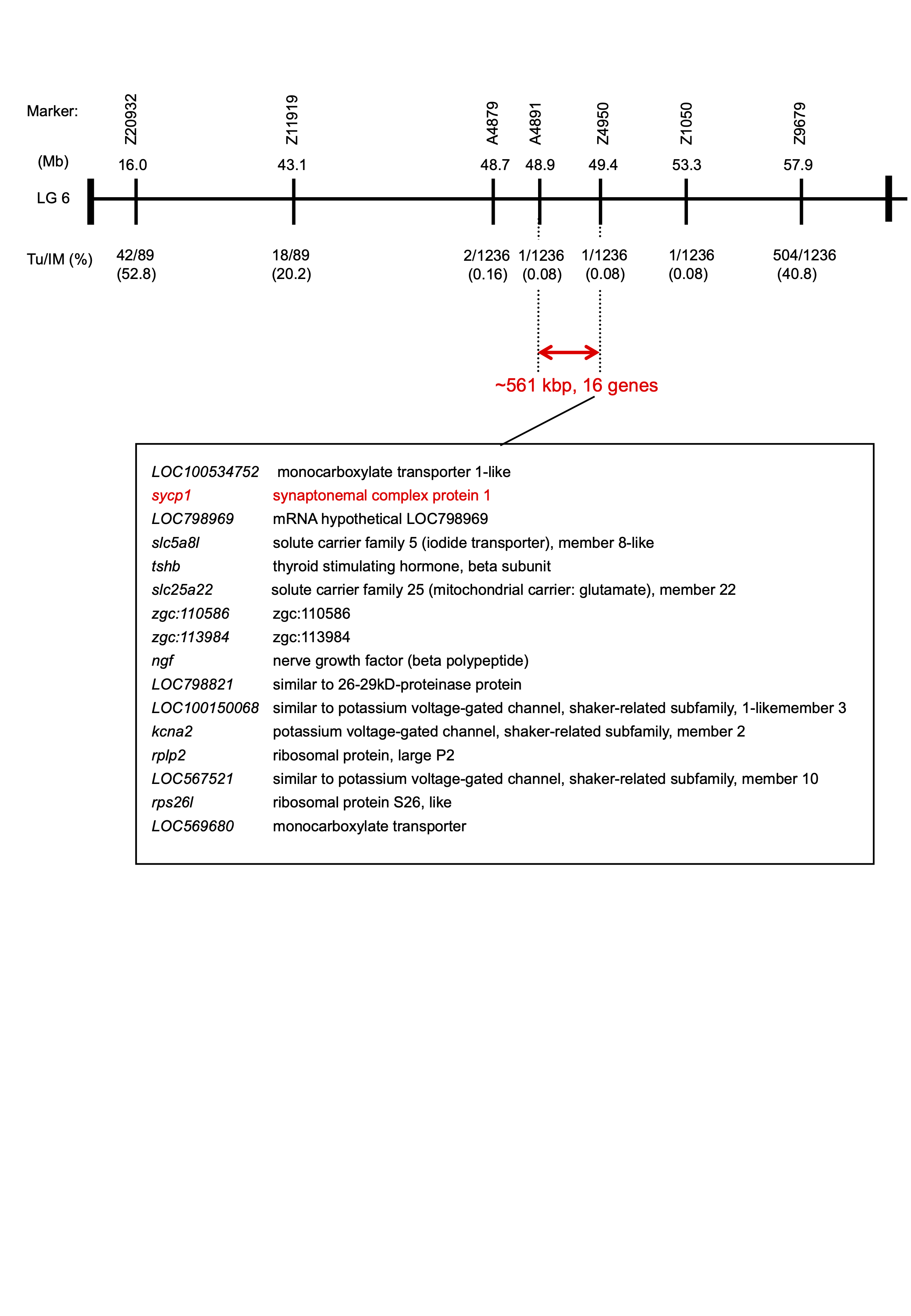

Supplement: Supplementary Figure 1 — Mapping of the isa mutation with SSLP markers. The names of the SSLP markers used and their locations on chromosome 6 (in Mb, the Zv9 zebrafish reference genome) are shown with mapping results. Tu: Tubingen alleles associated with the ENU-mutated line)/IM: inbred India alleles associated with the wild-type line used for backcrossing. Sixteen genes found in an ∼561-kbp region between markers A4891 and z4950 are listed below. [file Image_1.TIFF]

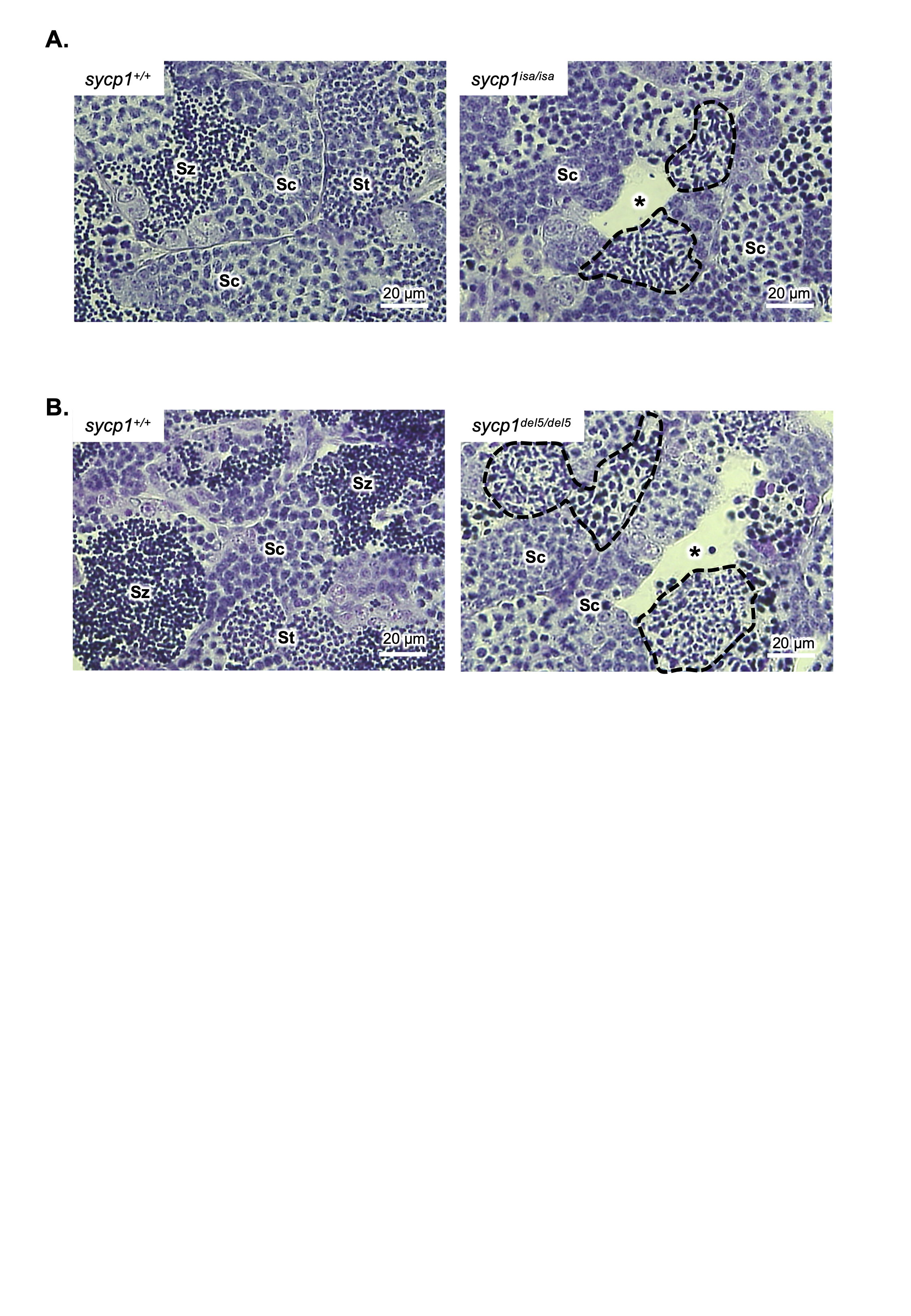

Supplement: Supplementary Figure 2 — HE-stained sections of sycp1+/+, sycp1isa/isa and sycp1del5/del5 testes. (A) images of sycp1+/+ and sycp1isa/isa testes from siblings at 4 mpf. (B) images of sycp1+/+ and sycp1del5/del5 testes from siblings at 4 mpf. Representative results of two individual fish are shown for each genotype. Sc: spermatocytes, St: spermatids, Sz: spermatozoa. Lumens with no spermatozoa (asterisks) and spermatocytes with irregular nuclei (inside broken lines) were observed in the sycp1isa/isa section, as previously reported (Saito et al., 2014), as well as in the sycp1del5/del5 section. [file Image_2.TIFF]

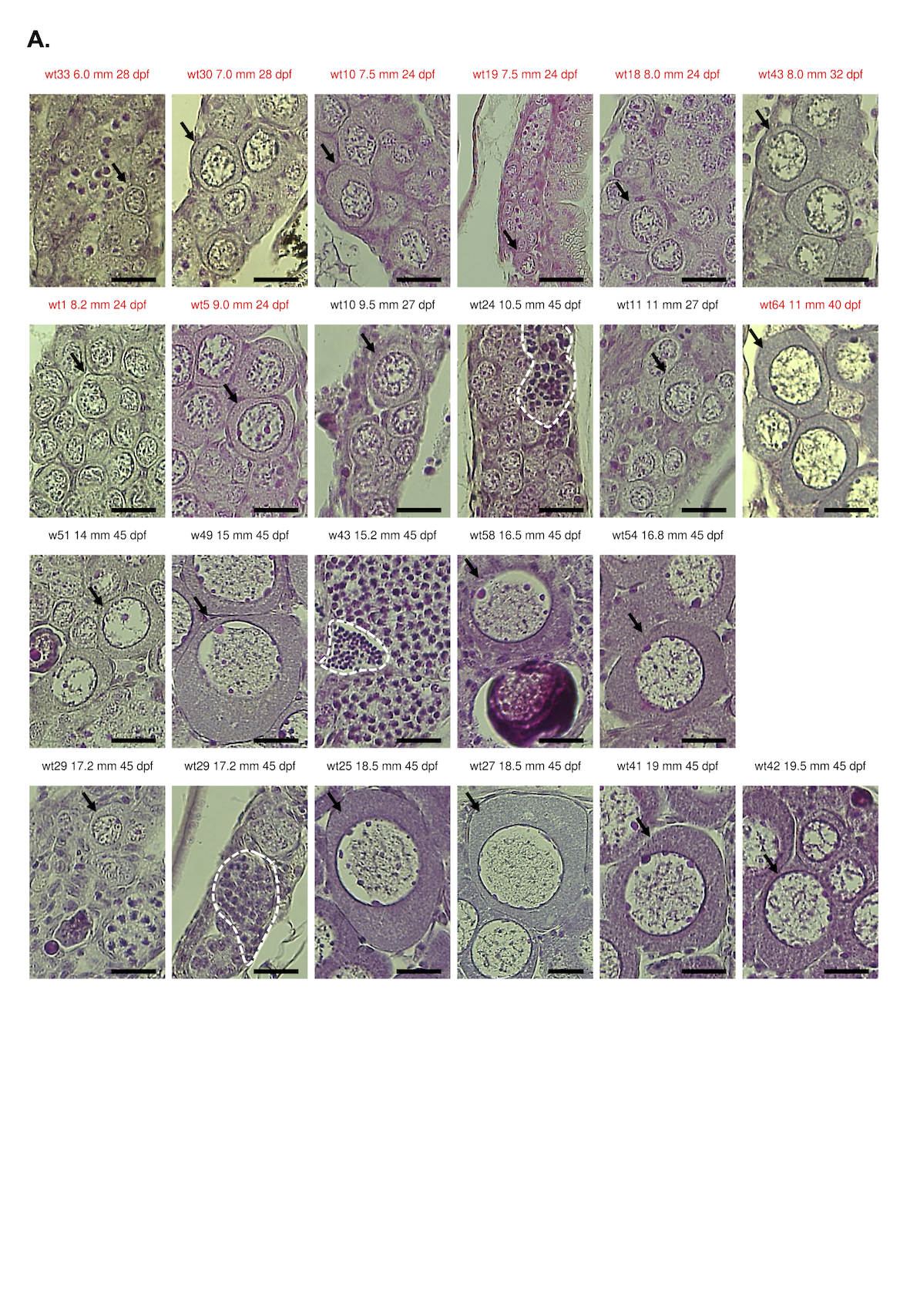

Supplement: Supplementary Figure 3 — HE-stained sections of juvenile gonads. Images of sycp1+/+ (A) and sycp1isa/isa (B) gonads are shown with fish ID, size (mm) and age (dpf). Samples were obtained from two different siblings indicated in red and black letters. The largest oocytes found on each section of gonads with oocytes are indicated with arrows. For gonads with spermatocysts, examples of spermatocysts are marked with white broken lines. Note that both oocytes and spermatocysts were observed in wt29 gonads (both images are shown here) and that neither oocytes nor spermatocysts were observed in mut14 gonads. These two fish were categorized as N.D. (not determined) in Figure 2C. Scale bars, 20 μm. [file Image_3.TIFF]

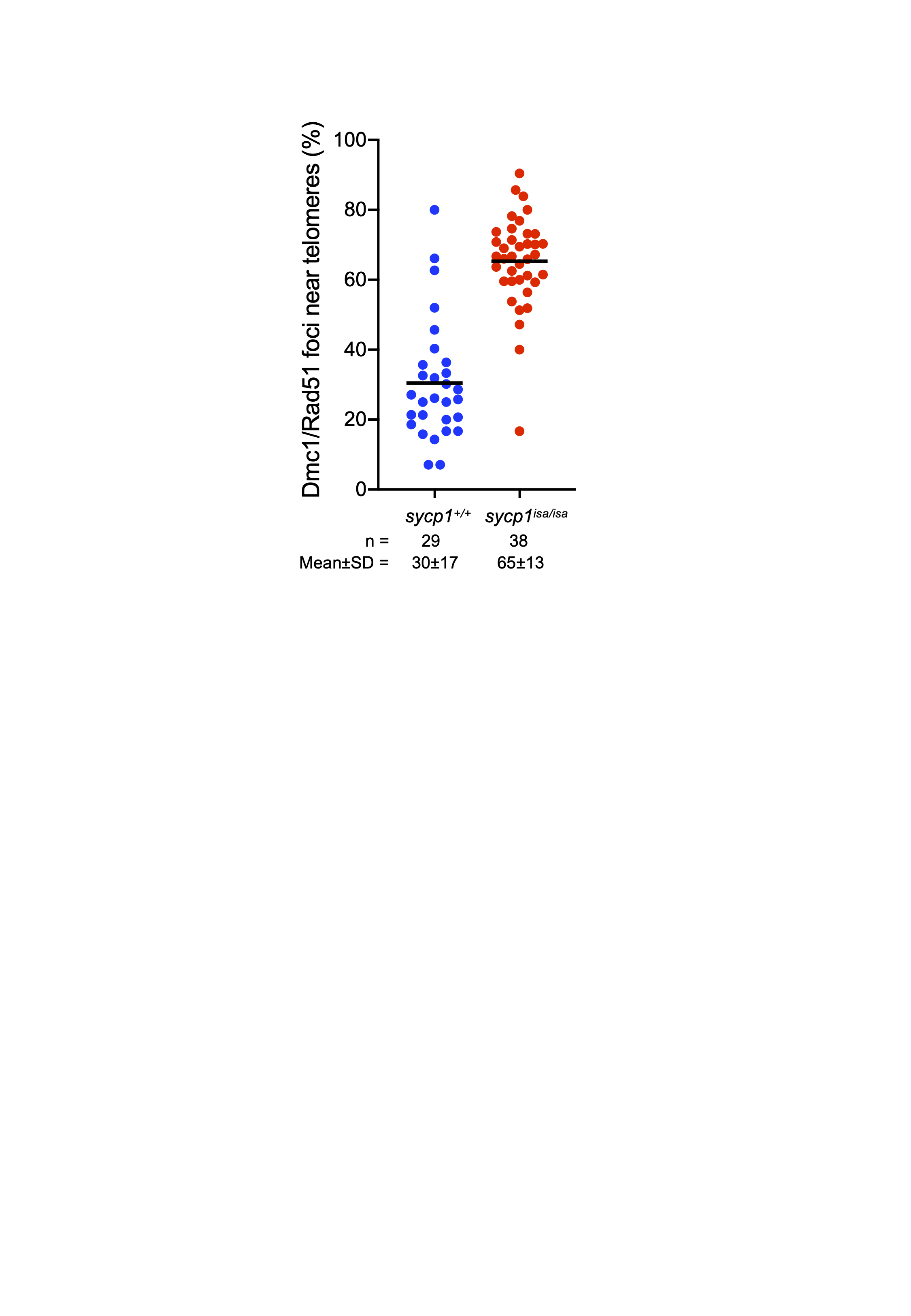

Supplement: Supplementary Figure 4 — Proportion of Dmc1/Rad51 foci in telomere-proximal regions. The number of Dmc1/Rad51 foci in telomere-proximal regions was counted in the MZ and MZ-like nuclei quantified for total Dmc1/Rad51 foci in Figure 6B. Proportion of Dmc1/Rad51 foci in telomere-proximal regions to total Dmc1/Rad51 foci in a nucleus was plotted for MZ sycp1+/+ and MZ-like sycp1isa/isa spermatocytes. [file Image_4.TIFF]

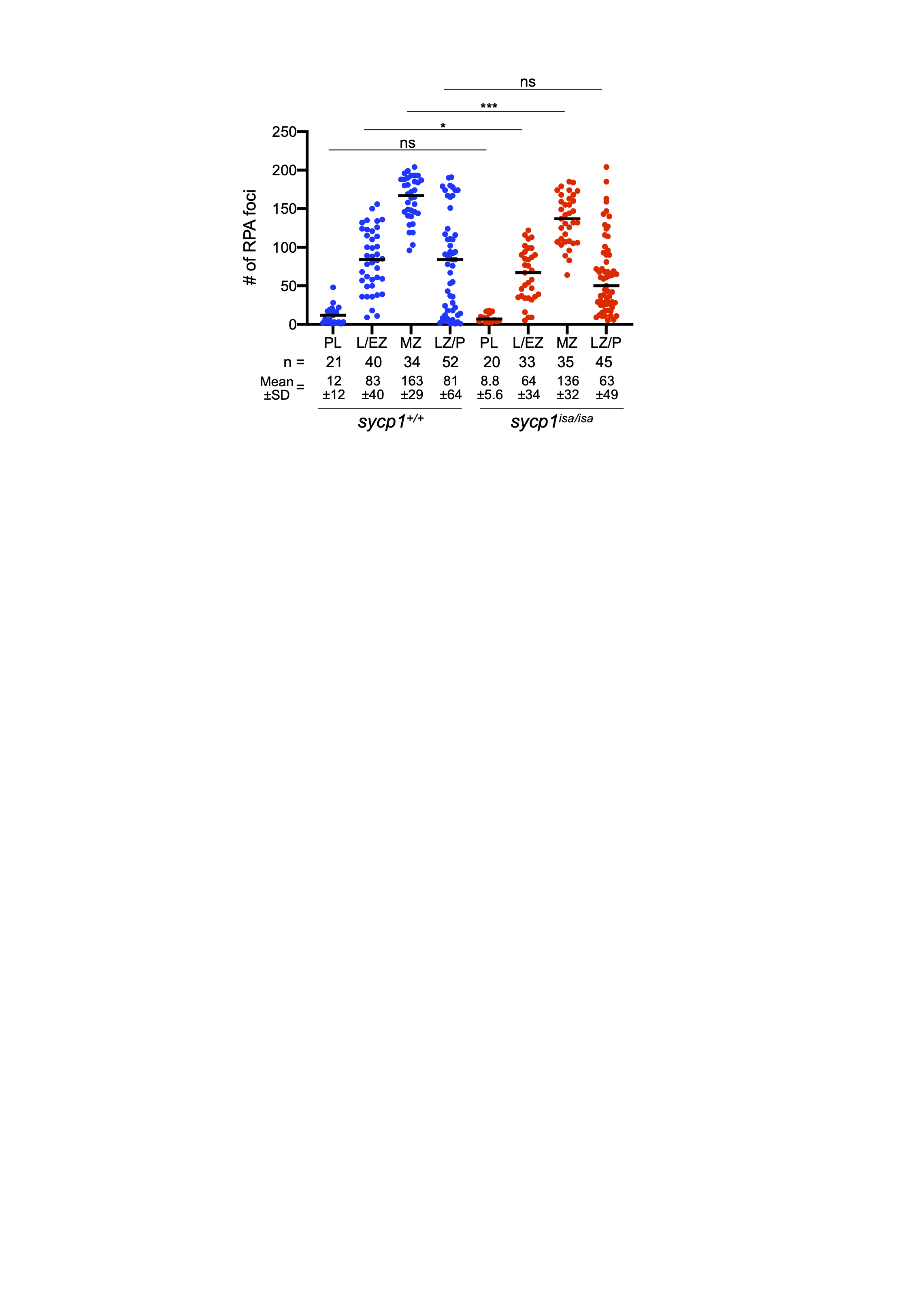

Supplement: Supplementary Figure 5 — Quantification of RPA foci in sycp1+/+ and sycp1isa/isa spermatocytes. The number of RPA foci was counted in the same nuclei quantified for RPA signal intensities, as shown in Figure 6D. Black bars indicate means. Statistical significance was examined by a two-tailed Mann-Whitney test (∗P < 0.1, ∗∗∗P < 0.001, ns, not significant; exact P value). [file Image_5.TIFF]

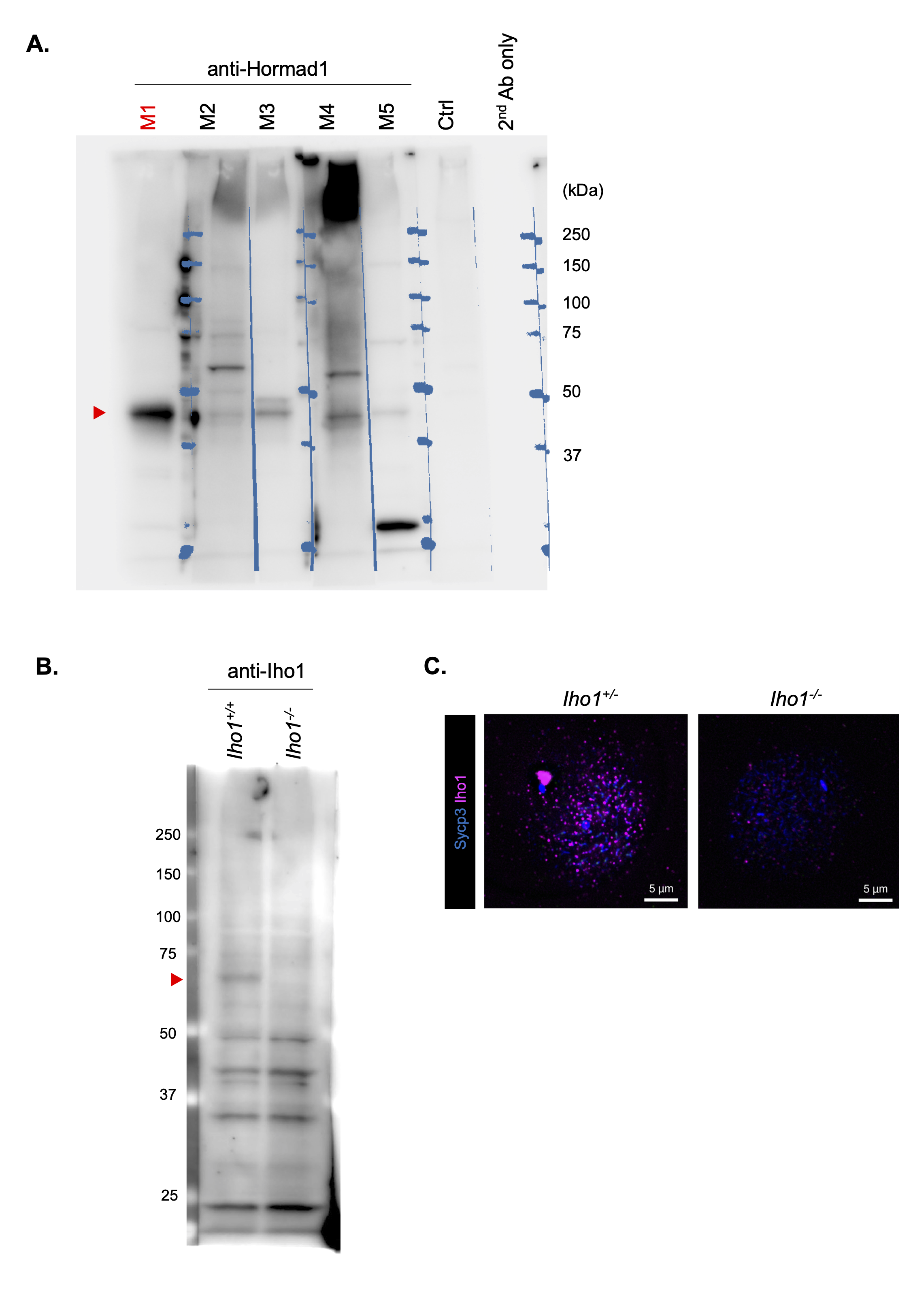

Supplement: Supplementary Figure 6 — Generation of antibodies against zebrafish Hormad1 and Iho1. (A) Western blotting of wild-type testis protein extracts with anti-zebrafish Hormad1 antisera obtained from five immunized mice (M1 to M5), unimmunized mouse serum (Ctrl) and no-serum control (2nd Ab only). The blot image is superposed with a binary image of colorimetric capture of molecular ladders (in blue). The band indicated with a red arrowhead is close to the predicted size of the full-length Hormad1 protein (41 kDa). Each well was loaded with 50 μg protein. M1 serum was used for immunostaining in this study (red). (B) Western blotting of protein extracts from Iho1+/+ and Iho1–/– testes with anti-zebrafish Iho1 antisera obtained from immunized mice. The predicted size of the full-length Iho1 protein is 61 kDa, and the band specific to the Iho1+/+ sample is indicated with a red arrow. Each well was loaded with 50 μg protein. (C) Coimmunostaining of Iho1 and Sycp3 on Iho1+/+ and Iho1–/– spermatocyte spreads. Iho1 signals were barely observed in Iho1–/– spermatocytes. [file Image_6.TIFF]
